# Supplementary material for: Phospholamban antisense oligonucleotides improve cardiac function in murine cardiomyopathy
Source: Nat Commun. 2021 Aug 30;12:5180. doi: 10.1038/s41467-021-25439-0 (PMC8405807; doi:10.1038/s41467-021-25439-0)
Supplement: Supplementary file 1 — Description of Additional Supplementary Files [file 41467_2021_25439_MOESM1_ESM.docx]

**Description of Additional Supplementary Files**

**File Name: Supplementary Data 1.**

**Description:** Source echocardiography data of the *Cspr3/Mlp^-/-^* studies.

EF = left ventricular ejection fraction, TP = Timepoint, ESV = left ventricular end systolic volume, EDV = left ventricular end diastolic volume.

**File Name: Supplementary Data 2.**

**Description:** List of primers used for the SYBR Green and Taqman assays

**File Name: Supplementary Data 3.**

**Description:** The exact P-values for all statistical test presented in the figures are provided.

**File Name: Supplementary Data 4 and 5.**

**Description:** The original R code utilized for the analyses of the RNA sequencing data.
