# Supplementary material for: Phospholamban antisense oligonucleotides improve cardiac function in murine cardiomyopathy
Source: Nat Commun. 2021 Aug 30;12:5180. doi: 10.1038/s41467-021-25439-0 (PMC8405807; doi:10.1038/s41467-021-25439-0)
Supplement: Supplementary file 9 — Reporting Summary [file 41467_2021_25439_MOESM9_ESM.pdf]

nature research

Corresponding author(s): Daniela Später, Ralph Knoell, Adam Mullick, Peter van der Meer

Last updated by author(s): Jul 2, 2021

Reporting Summary

Nature Research wishes to improve the reproducibility of the work that we publish. This form provides structure for consistency and transparency in reporting. For further information on Nature Research policies, see our Editorial Policies and the Editorial Policy Checklist.

Statistics

For all statistical analyses, confirm that the following items are present in the figure legend, table legend, main text, or Methods section.

n/a	Confirmed	
☐	☒	The exact sample size (*n*) for each experimental group/condition, given as a discrete number and unit of measurement
☐	☒	A statement on whether measurements were taken from distinct samples or whether the same sample was measured repeatedly
☐	☒	The statistical test(s) used AND whether they are one- or two-sided<br>*Only common tests should be described solely by name; describe more complex techniques in the Methods section.*
☒	☐	A description of all covariates tested
☐	☒	A description of any assumptions or corrections, such as tests of normality and adjustment for multiple comparisons
☐	☒	A full description of the statistical parameters including central tendency (e.g. means) or other basic estimates (e.g. regression coefficient) AND variation (e.g. standard deviation) or associated estimates of uncertainty (e.g. confidence intervals)
☐	☒	For null hypothesis testing, the test statistic (e.g. *F*, *t*, *r*) with confidence intervals, effect sizes, degrees of freedom and *P* value noted<br>*Give P values as exact values whenever suitable.*
☒	☐	For Bayesian analysis, information on the choice of priors and Markov chain Monte Carlo settings
☒	☐	For hierarchical and complex designs, identification of the appropriate level for tests and full reporting of outcomes
☒	☐	Estimates of effect sizes (e.g. Cohen's *d*, Pearson's *r*), indicating how they were calculated

Our web collection on statistics for biologists contains articles on many of the points above.

Software and code

Policy information about availability of computer code

Data collection	PharmLab (V6.6, AstraZeneca R&D Mölndal, Sweden) was used for ECG and hemodynamic data collection in the rat study and ventricular pressures were measured via a pressure catheter (1.4F, SPR-847, Millar Instruments, Houston, Texas, US). The SA instruments platform (SA Instruments, Inc., USA) was used for MRI data collection in the rat study. Cobas 6000 (c502) chemistry platform (Roche, Mannheim, Germany) and an clinical analyzer (Olympus, Center Valley, PA) were used for plasma ALT and AST measurements. The Vevo 2100 System (Visualsonics, Canada) was utilized for echocardiography in the Cspr3/Mlp-/- studies. ParaVison 4.0 and IntraGate software (Bruker Biospin GmH) were used for cine MR acquisition and reconstruction in the PLN R14del studies. A PowerLab 8/30 data acquisition device (model ML870, ADInstruments, Australia) and an animal Bio Amp biological potential amplifier (model ML136, ADInstruments) were utilized to record ECG tracings.
Data analysis	For RNA sequencing: reads were aligned to mouse reference genome (mm10) using STAR 2.4.2a and readcount analysis was performed using htseq-count 0.6.1. Differential expression analysis was performed using DeSeq2 1.24.0. Gene set enrichment analysis was performed using Fgsea 1.10.0 and MsigDB v6.2. CVI42 (version 5.6.6, Circle Cardiovascular Imaging, Canada) was used for the determination of the LV end-diastolic volume, LV end-systolic volume, stroke volume, and ejection fraction from the MRI images of the PLN R14d/d study. LabChart Pro software version 8 (ADInstruments) was used for ECG analyses. Segment (Medviso AB, Sweden) was used for MRI analyses of the rat study. All other analyses were carried out using ImageJ version 2.0.0, GraphPad Prism software version 8.02 (GraphPad Software Inc.), STATA version SE 16.0 (StataCorp), or MatLab (The Mathworks, Natick, MA, USA)

For manuscripts utilizing custom algorithms or software that are central to the research but not yet described in published literature, software must be made available to editors and reviewers. We strongly encourage code deposition in a community repository (e.g. GitHub). See the Nature Research guidelines for submitting code & software for further information.

Data

Policy information about availability of data

All manuscripts must include a data availability statement. This statement should provide the following information, where applicable:

- Accession codes, unique identifiers, or web links for publicly available datasets
- A list of figures that have associated raw data
- A description of any restrictions on data availability

The data supporting the findings from this study are available in the article and the supplementary information. RNA-sequencing data are available in the GEO under accession number GSE151156 at https://www.ncbi.nlm.nih.gov/geo/query/acc.cgi?acc=GSE151156. Any remaining raw data will be available from the corresponding author upon reasonable request. Source data are provided with this paper.

Field-specific reporting

Please select the one below that is the best fit for your research. If you are not sure, read the appropriate sections before making your selection.

☒ Life sciences ☐ Behavioural & social sciences ☐ Ecological, evolutionary & environmental sciences

For a reference copy of the document with all sections, see nature.com/documents/nr-reporting-summary-flat.pdf

Life sciences study design

All studies must disclose on these points even when the disclosure is negative.

Sample size	For R14del studies: for the endpoint of PLN protein downregulation we expected a 70% reduction based on previous experiments with the PLN-ASO in other mouse models. With a standard deviation of 20% and a minimal power of 0.8 and an alpha of 0.05 this resulted in 4 animals per group. For lifespan, we hypothesized that the PLN-ASO would be able to increase the maximum life span of 8.25 ± 0.5 weeks with 10% to 9.1 weeks. This resulted in a minimum of 5 animals per group, based on a power of 0.8 and an alpha of 0.05.<br>For the Cspr3/Mlp-/- study: Very little information was available to base a sample size calculation on. We used left-ventricular end-diastolic dimension (LVEDD) at 2 months as reported in Minamisawa et al. (1999) (table 1) for double KO mice compared to WT to perform a rough sample size calculation. Assuming that LVEDD is approximately normally distributed with a standard deviation of 0.34, we can detect a difference of 0.4 mm between two groups using a one-sided t-test at a significance level of 0.05 with 80% power using at least 10 animals per group.<br>For the rat MI study: Using a linear statistical model and one-sided contrasts of the estimated means the hypothesis was to detect at least one difference with the PLN-ASO dose 1 and 2 treatment groups (both with mean=5 and sd=2.5) and the Control-ASO and at least one difference with the Control-PBS (both with mean=3.5, sd=2.5). The p-values are adjusted by Holm's method and all group sizes are set to be equal at n=7 based on a power of 0.8 and an alpha of 0.05.
Data exclusions	One mouse in the R14del study was terminated before study completion because of severe underweight, in retrospect already present at study start. In the rat MI study two rats were excluded from the PLN ASO 50mg/kg group, one due to an incomplete MRI dataset after the second measurement, and a second rat died before the final MRI scan.
Replication	Replication consisted of three repeated Cspr3/Mlp knockout studies, which yielded similar results. For the other in vivo studies, no replication was performed. Experimental procedures such as western blots, qPCRs etc. were replicated on a individual basis and yielded similar results.
Randomization	Randomization was performed for every in vivo study. R14del mice were randomized based on sex. MLP knockout mice were randomized based on baseline cardiac function (left ventricular ejection fraction), body weight, age, and gender. Rats were randomized based on plasma cTnI levels one day post infarction, visual infarct size score, body weight, and heart rate at the baseline (6 weeks post MI).
Blinding	For all in vivo studies: mice handling, experimental in vivo procedures, symptoms assessment and other assessments were performed by blinded investigators. Experimental in vitro follow-up studies were performed blinded, western blots were repeated unblinded to allow for better visualization for publication.

Reporting for specific materials, systems and methods

We require information from authors about some types of materials, experimental systems and methods used in many studies. Here, indicate whether each material, system or method listed is relevant to your study. If you are not sure if a list item applies to your research, read the appropriate section before selecting a response.

Materials & experimental systems

n/a	Involved in the study	
	x	Antibodies
x		Eukaryotic cell lines
x		Palaeontology and archaeology
	x	Animals and other organisms
x		Human research participants
x		Clinical data
x		Dual use research of concern

Methods

n/a	Involved in the study	
x		ChIP-seq
x		Flow cytometry
x		MRI-based neuroimaging

Antibodies

Antibodies used	anti-PLN 2D12 monoclonal (ThermoFisher: #MA3-922 1:1000)<br>anti-SERCA2 (Abcam ab150435 1:1000-1:5000 or ThermoFisher: #MA3-919 1:1000)<br>anti-phospho-PLN (Cell Signaling: #8496 1:1000)<br>anti-GAPDH-HRP (Invitrogen MAS-15738 1:2000 or 1:35.000)<br>anti-cardiac troponin I (Abcam 47003 1:100)<br>anti-Caveolin-3 (Abcam 2912 1:50)<br>Alexa Fluor 488 goat anti rabbit (ab150077, Abcam, 1:100)
Validation	All used antibodies concern commercially available and extensively tested antibodies. Only the anti-PLN 2D12 antibody was validated in PLN knock-out heart tissue (supplementary figure 17). For the other used antibodies no further validation is provided in our in our manuscript, but we refer to the website of the manufacturer for validation experiments and extensive lists of publications.<br><br>ThermoFisher MA3-922: https://www.thermofisher.com/antibody/product/Phospholamban-Antibody-clone-2D12-Monoclonal/MA3-922<br>Abcam ab150435: https://www.abcam.com/serca2-atpase-antibody-epr9392-ab150435.html<br>ThermoFisher MA3-919: https://www.thermofisher.com/antibody/product/SERCA2-ATPase-Antibody-clone-2A7-A1-Monoclonal/MA3-919<br>Cell signaling 8496: https://www.cellsignal.com/products/primary-antibodies/phospho-phospholamban-ser16-thr17-antibody/8496<br>Invitrogen MAS-15738: https://www.thermofisher.com/antibody/product/GAPDH-Loading-Control-Antibody-clone-GA1R-Monoclonal/MA5-15738<br>Abcam 47003: https://www.abcam.com/cardiac-troponin-i-antibody-ab47003.html<br>Abcam 2912: https://www.abcam.com/caveolin-3-antibody-caveolae-marker-ab2912.html<br>Abcam 150077: https://www.abcam.com/goat-rabbit-igg-hl-alexa-fluor-488-ab150077.html

Animals and other organisms

Policy information about studies involving animals; ARRIVE guidelines recommended for reporting animal research

Laboratory animals	All laboratory animals used for this report were housed on a 12 h light / 12 h dark cycle with ad libitum access to chow and water. Rats and mice for the Cspr3/Mlp experiments were housed at an ambient temperature of ~21-22C and 50% humidity, mice for the PLN R14del studie were housed at an ambient temperature of 20-24C and 45-65% humidity.<br><br>For adult mouse cardiomyocyte isolation used for the mouse PLN ASO screen: C57BL6/N mice, male, 6-8 weeks of age.<br><br>For PLN R14del studies: C57BL6/N mice, either wild-type or with an introduced c.40-42del AGA mutation. Both males and females at the age of 3-26 weeks, or the age of 3 days for neonatal cardiomyocyte isolation and culture.<br><br>For Cspr3/Mlp studies: Cspr3/Mlp+/+ or Cspr3/Mlp-/- mice in C57BL6/N background were used, both males and females at the age of 16-41 weeks.<br><br>For rat myocardial infarction studies: male Lewis rats at the age of 8 weeks were used at study initiation for LAD ligation or sham procedure.
Wild animals	The study did not involve wild animals
Field-collected samples	The study did not include field-collected samples
Ethics oversight	All experimental protocols were approved by the local Animal Ethical Committee.<br>In the Netherlands for the PLN R14del experiments by the "Centrale Commissie Dierproeven [CCD] and Animal Welfare Body (permit numbers: AVD10500201583, IVD1583-02-001 and IVD1583-02-006<br>For the animals studies in Sweden by the local Animal Welfare Body, permit: 86-2015 for rat MI study and 43-15 for Cspr3/Mlp-/- study 1 and 2 in Sweden.<br>For the animal study in the USA by the local Animal Welfare Body, permit: P-0308-100113.<br>All animal experiments were performed conform the ARRIVE guidelines.

Note that full information on the approval of the study protocol must also be provided in the manuscript.
