# Supplementary material for: Phospholamban antisense oligonucleotides improve cardiac function in murine cardiomyopathy
Source: Nat Commun. 2021 Aug 30;12:5180. doi: 10.1038/s41467-021-25439-0 (PMC8405807; doi:10.1038/s41467-021-25439-0)
Supplement: Supplementary file 10 — Supplementary Information [file 41467_2021_25439_MOESM10_ESM.pdf]

Supplementary Information

Phospholamban Antisense Oligonucleotides Improve Cardiac Function in Murine Cardiomyopathy

Authors: Niels Grote Beverborg[1¤], Daniela Später[2,3,¤,*], Ralph Knöll[2,3,¤,*], Alejandro Hidalgo[2,3,4], Steve T. Yeh[5], Zaher Elbeck[3], Herman H.W. Silljé[1], Tim R. Eijgenraam[1], Humam Siga[3], Magdalena Zurek[2], Malin Palmér[2,6], Susanne Pehrsson[2], Tamsin Albery[2], Nils Bomer[1], Martijn F. Hoes[1], Cornelis J. Boogerd[7], Michael Frisk[8], Eva van Rooij[7], Sagar Damle[5], William E. Louch[8], Qing-Dong Wang[2], Regina Fritsche-Danielson[2], Kenneth R. Chien[3,9], Kenny Hansson[2], Adam E. Mullick[5,*,#], Rudolf A. de Boer[1,#], Peter van der Meer[1,*,#]

¤ = These authors contributed equally

# = These authors jointly supervised this work

* = corresponding author

Institutions: [1]University of Groningen, University Medical Center Groningen, Department of Cardiology, Groningen, The Netherlands [2]Bioscience Cardiovascular, Research and Early Development, Cardiovascular, Renal and Metabolism (CVRM), BioPharmaceuticals R&D, AstraZeneca, Gothenburg, Sweden, [3]Integrated Cardio Metabolic Center (ICMC), Karolinska Institutet, Huddinge, Sweden, [4]present address: Murdoch Children's Research Institute (MCRI), Flemington, Melbourne, Australia, and Department of Paediatrics, University of Melbourne, Melbourne, Australia, [5]Ionis Pharmaceuticals, Carlsbad, CA, USA, [6]Laboratory of Experimental Biomedicine, Core Facilities, Sahlgrenska Academy, Gothenburg University, Göteborg, Sweden, [7]Hubrecht Institute, Royal Netherlands Academy of Arts and Sciences (KNAW) and University Medical Center Utrecht, Department of Molecular Cardiology, Utrecht, The Netherlands, [8]Institute for Experimental Medical Research, Oslo University Hospital and KG Jebsen Center for Cardiac Research, University of Oslo, Norway, [9]Department of Cell and Molecular Biology (CMB), Karolinska Institute, Stockholm, Sweden. [¤]email: daniela.spaeter@astrazeneca.com, Ralph.Knoell@AstraZeneca.com / ralph.knoell@ki.se, AMullick@ionisph.com, p.van.der.meer@umcg.nl.

Supplementary Information: 22

Supplementary Figure 1. PLN protein is downregulated to <50% of PBS without relevant ALT or AST increases for the duration of 6 weeks after 3 administrations of ASO#27 or ASO#26_C

a

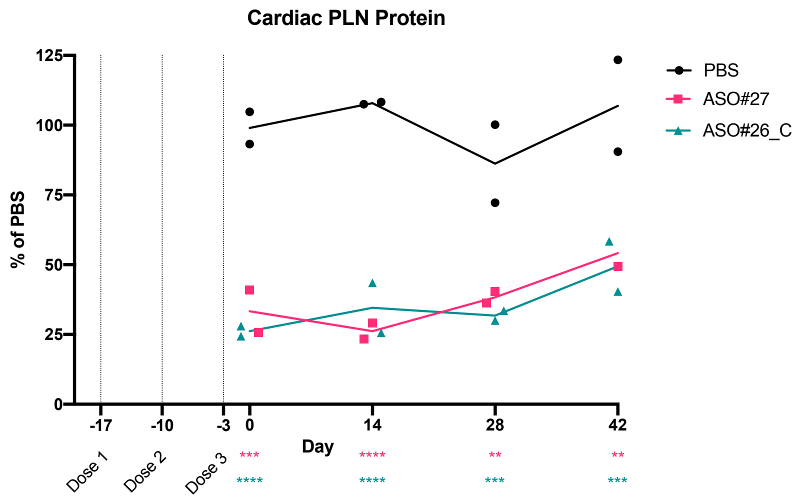

b

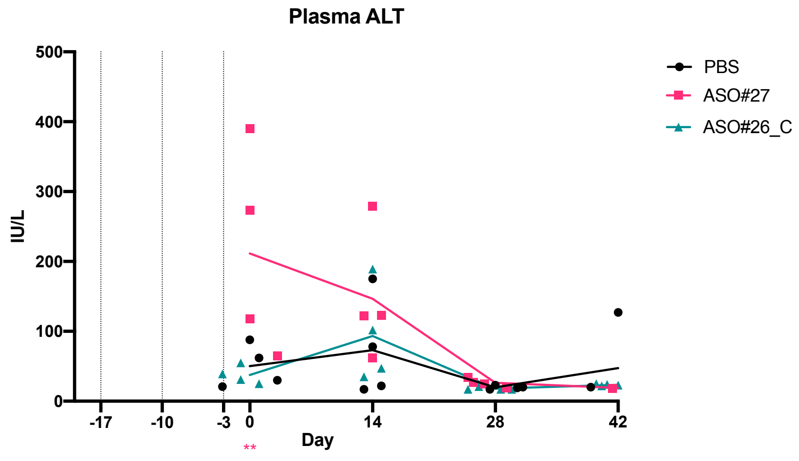

c

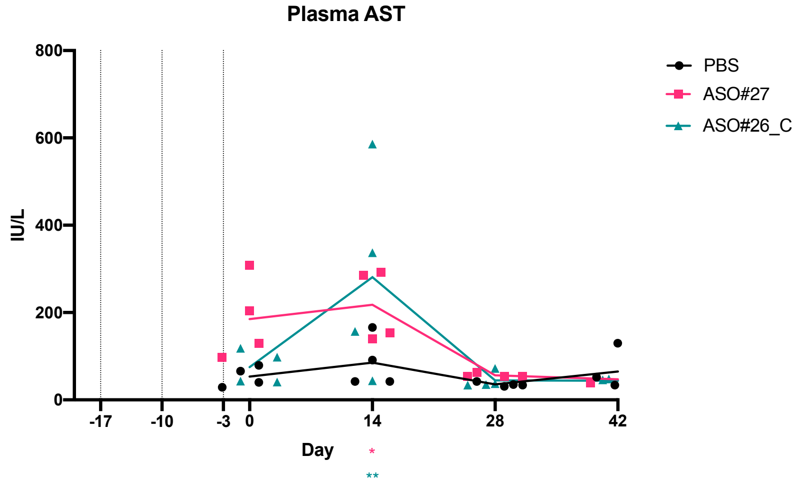

Supplementary Figure 1. PLN protein is downregulated to <50% of PBS without relevant ALT or AST increases for the duration of 6 weeks after 3 administrations of ASO#27 or ASO#26_C
Comparison of treatment effect and potential side effects on plasma aspartate transaminase (AST) and alanine transaminase (ALT) between ASO#27 and ASO#26_C. Mice received 3 doses of the PLN-ASO of either 50mg/kg (ASO#26_C) or 100mg/kg (ASO#27), analyses were performed at day 0, 14, 28 and 42. (a) Western blot quantification of PLN protein levels normalized against PBS injection control. (b and c) Plasma ALT and AST levels. No relevant increases compared to PBS are detected. Two-way analysis of variance was used for analyses, with PBS treated animals as the reference group in multiple comparison analysis. Asterix denotes significance level compared to PBS for ASO#27 (pink) and ASO#26_C (green) with: *P-value<0.05, **P-value<0.01, ***P-value<0.001 and ****P-value<0.0001. Single values are depicted and lines represent the mean.

Supplementary Figure 2. Full western blots of PLN R14del$^{\Delta/\Delta}$ study showing PLN protein expression over the course of the study

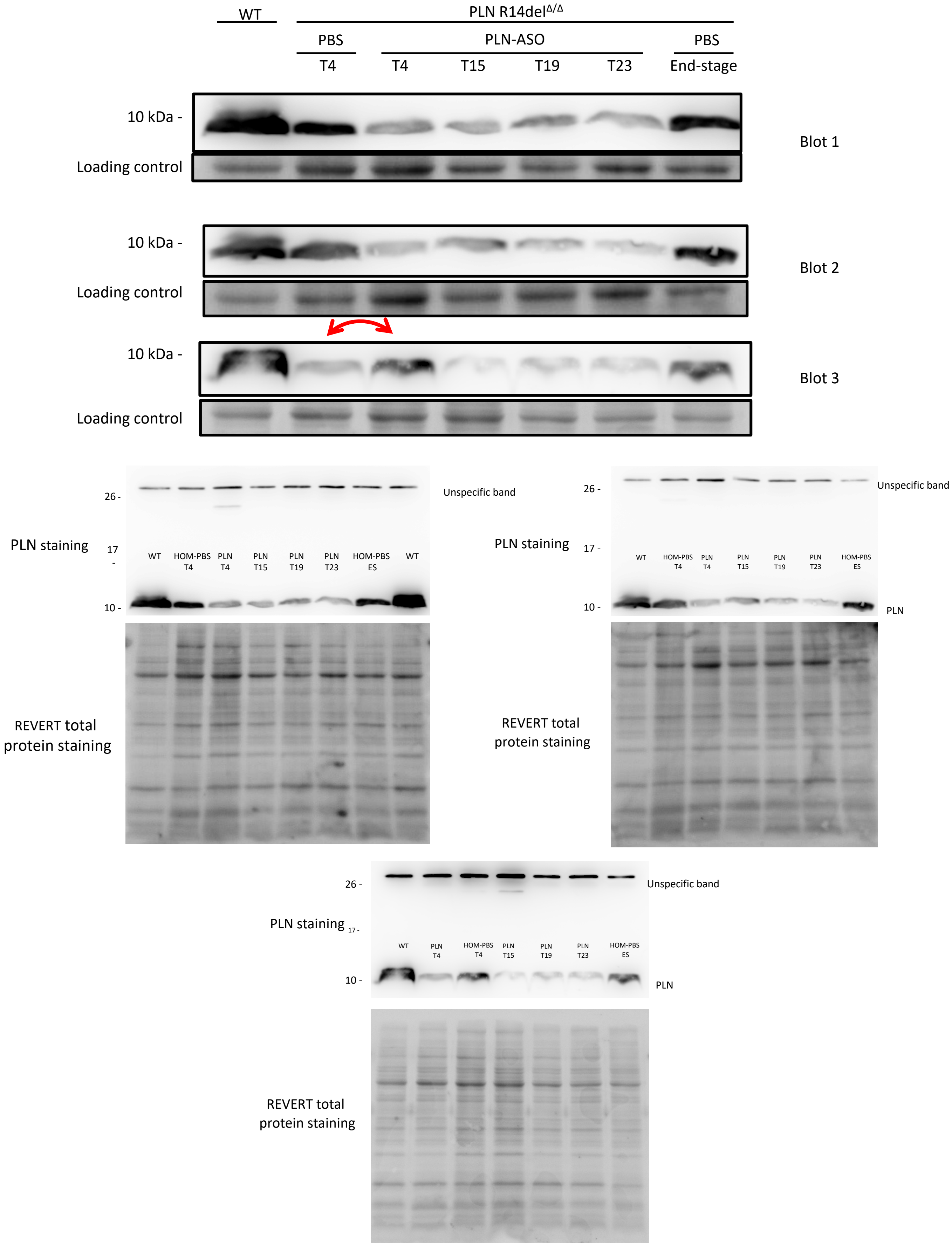

Supplementary Figure 2. Full western blots of PLN R14del$^{\Delta/\Delta}$ study showing PLN protein expression over the course of the study

Full images of the western blots performed in the PLN R14del$^{\Delta/\Delta}$ study, stained for PLN and with total protein staining (Revert 700) as a loading control. Protein lysates were obtained with RIPA, the remaining pellet was dissolved in Urea, both fractions were merged in ratio. Samples were boiled resulting in the degradation of all oligomers into monomers of +/- 7kDa. Please note that for blot 3, PBS T4 and PLN-ASO T4 were accidentally switched compared to the other 2 blots (red arrows).

Supplementary Figure 3. Full western blots of PLN R14del$^{\Delta/\Delta}$ study showing PLN and SERCA2 protein expression at T4 / age of 7 weeks (RIPA blot)

RIPA blot
PLN staining

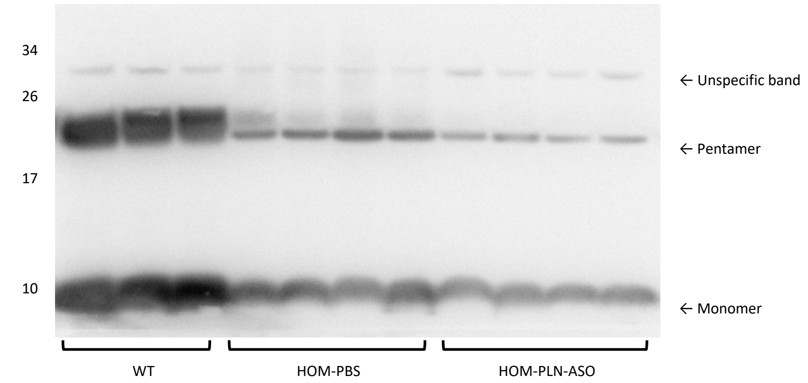

SERCA2 staining

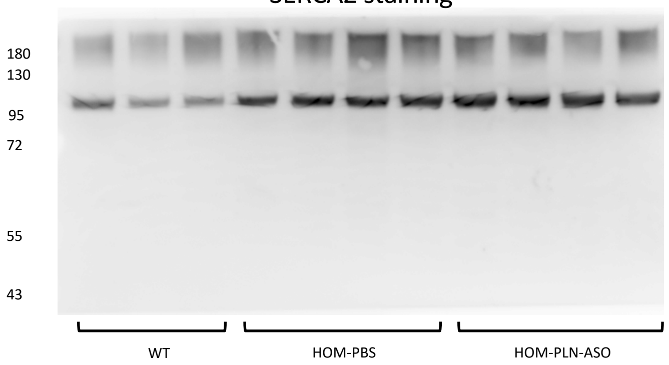

REVERT total protein

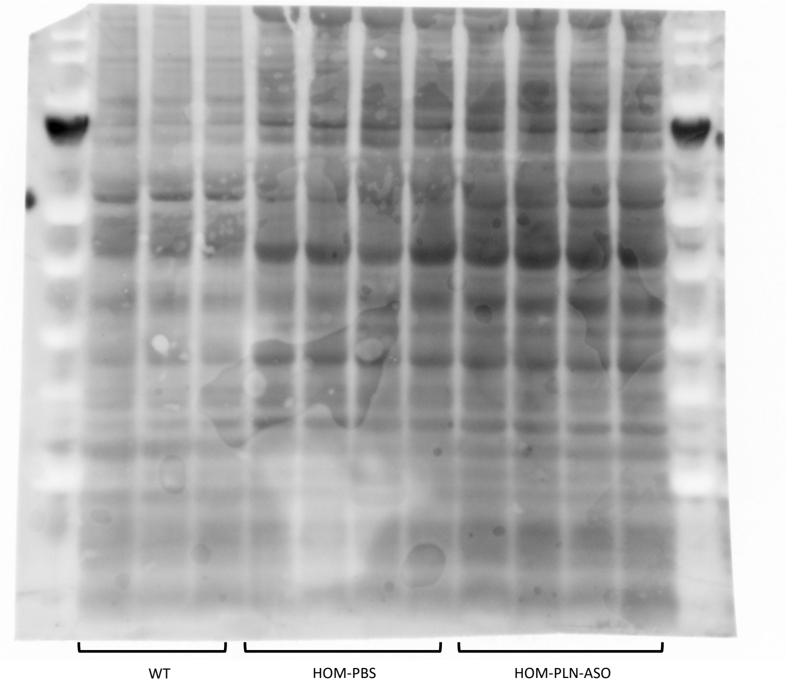

Supplementary Figure 3. Full western blots of PLN R14del$^{\Delta/\Delta}$ study showing PLN and SERCA2 protein expression at T4 / age of 7 weeks (RIPA blot)

Full images of the western blots performed in the PLN R14del$^{\Delta/\Delta}$ study on protein isolated with RIPA buffer at treatment week 4 (T4) or the age of 7 weeks. Samples were not boiled, thus keeping intact oligomers of PLN. Blots are stained for PLN, SERCA2 and REVERT total protein staining as a loading control.

Supplementary Figure 4. Full western blots of PLN R14del$^{\Delta/\Delta}$ study showing aggregated PLN protein at T4 / age of 7 weeks (Urea blot)

Urea blot
PLN staining

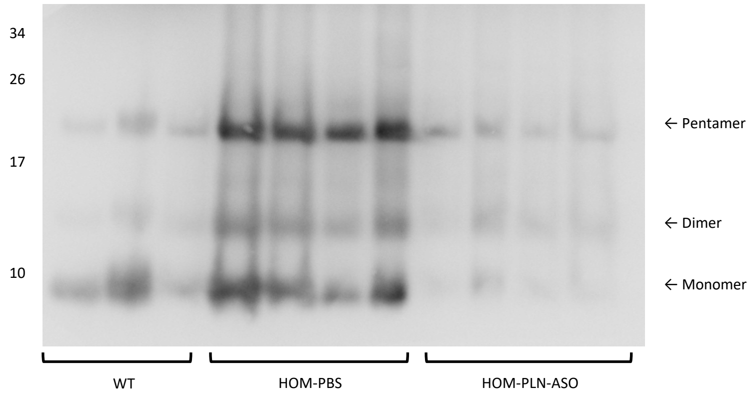

SERCA2 staining

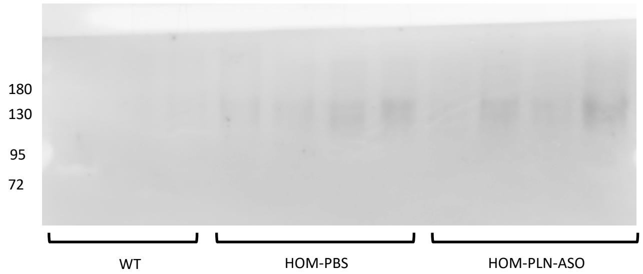

REVERT total protein

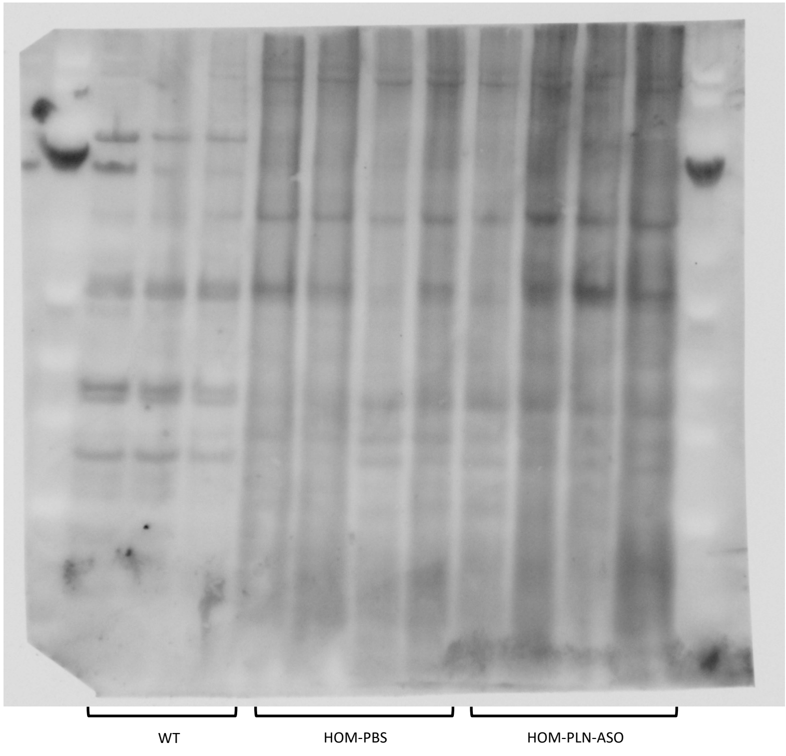

Supplementary Figure 4. Full western blots of PLN R14del$^{\Delta/\Delta}$ study showing aggregated PLN protein at T4 / age of 7 weeks (Urea blot)

Full images of the western blots performed in the PLN R14del$^{\Delta/\Delta}$ study on protein isolated from the RIPA pellet using Urea at treatment week 4 (T4) or the age of 7 weeks. Samples were not boiled, thus keeping intact oligomers of PLN. Blots are stained for PLN, SERCA2 and REVERT total protein staining as a loading control.

Supplementary Figure 5. PLN protein levels are decreased in PLN-ASO treated PLN R14del$^{\Delta/\Delta}$ mice, especially oligomers solvable in urea representing the aggregated protein fraction

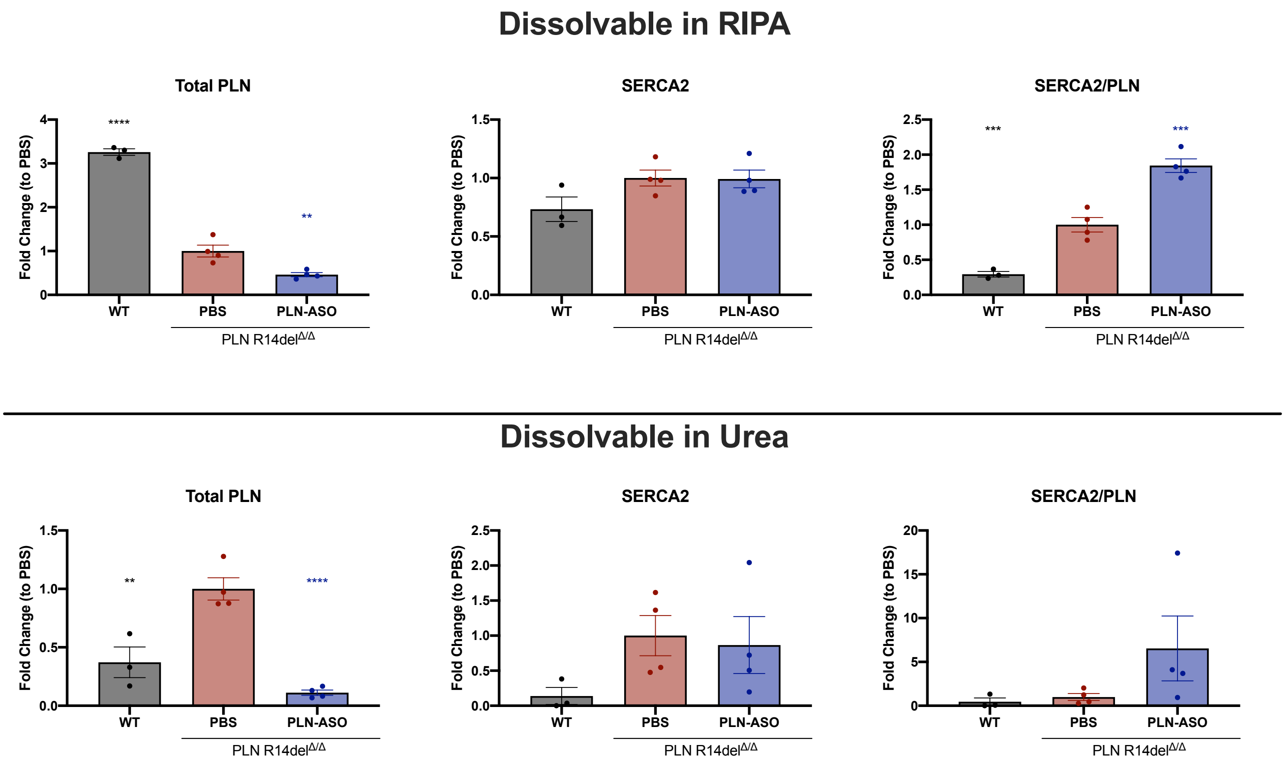

Supplementary Figure 5. PLN protein levels are decreased in PLN-ASO treated PLN R14del$^{\Delta/\Delta}$ mice, especially oligomers solvable in urea representing the aggregated protein fraction. Quantification of the western blots of Supplementary Figures 3 and 4 for SERCA2a and total PLN. One-way analyses of variance was used for analyses, with PBS treated animals as the reference group in multiple comparison analyses. Asterix denotes significance level compared to PBS with: *P-value<0.05, **P-value<0.01, ***P-value<0.001 and ****P-value<0.0001. Single values are depicted and error bars represent standard error of the mean (SEM). n=3 for wild-type, n=4 for PBS and PLN-ASO groups.

Supplementary figure 6. Heart failure related gene expressions are downregulated in PLN R14del$^{\Delta/\Delta}$ mice upon PLN-ASO treatment at T4 / age of 7 weeks

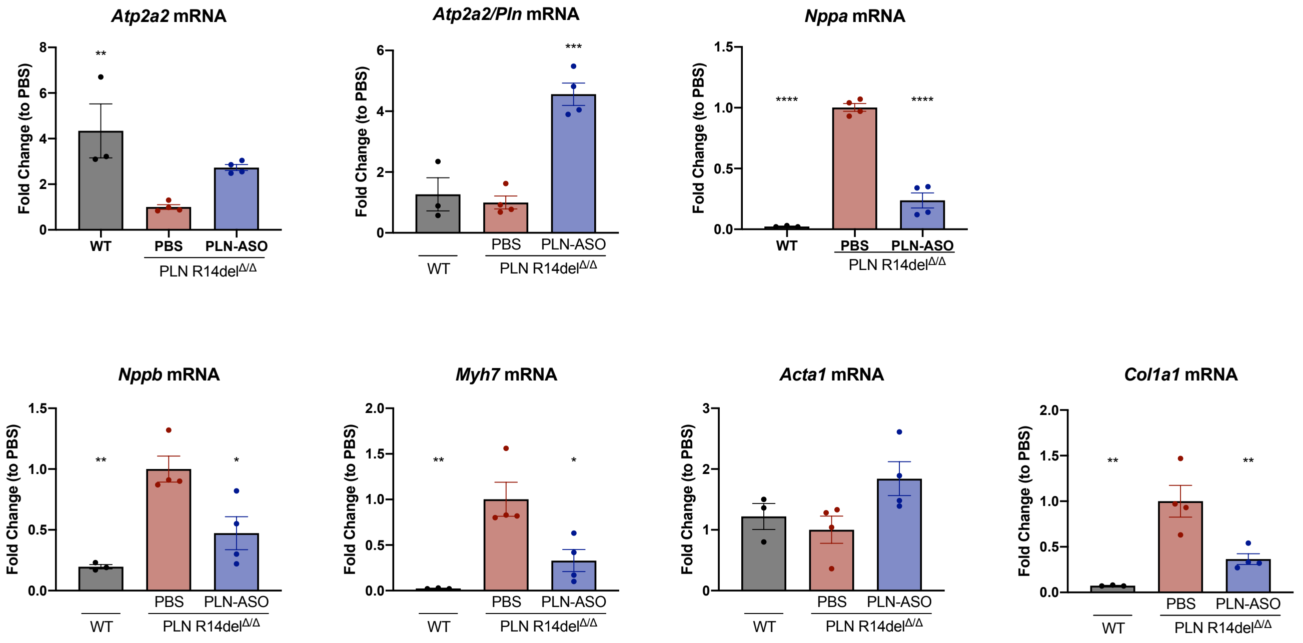

Supplementary Figure 6. Heart failure related gene expressions are downregulated in PLN R14del$^{\Delta/\Delta}$ mice upon PLN-ASO treatment at T4 / age of 7 weeks

Gene expression analyses of left ventricular heart tissue from mice in the PLN R14del$^{\Delta/\Delta}$ study at treatment week 4 (T4) or the age of 7 weeks. One-way analysis of variance was used for analyses, with PBS treated animals as the reference group in multiple comparison analysis. Asterix denotes significance level compared to PBS with: *P-value<0.05, **P-value<0.01, ***P-value<0.001 and ****P-value<0.0001. Single values are depicted and error bars represent standard error of the mean (SEM). n=3 for wild-type, n=4 for PBS and PLN-ASO groups.

Supplementary Figure 7. No signs of increased heart rate or toxicity in PLN R14delΔ/Δ mice at T4/ 7 weeks of age

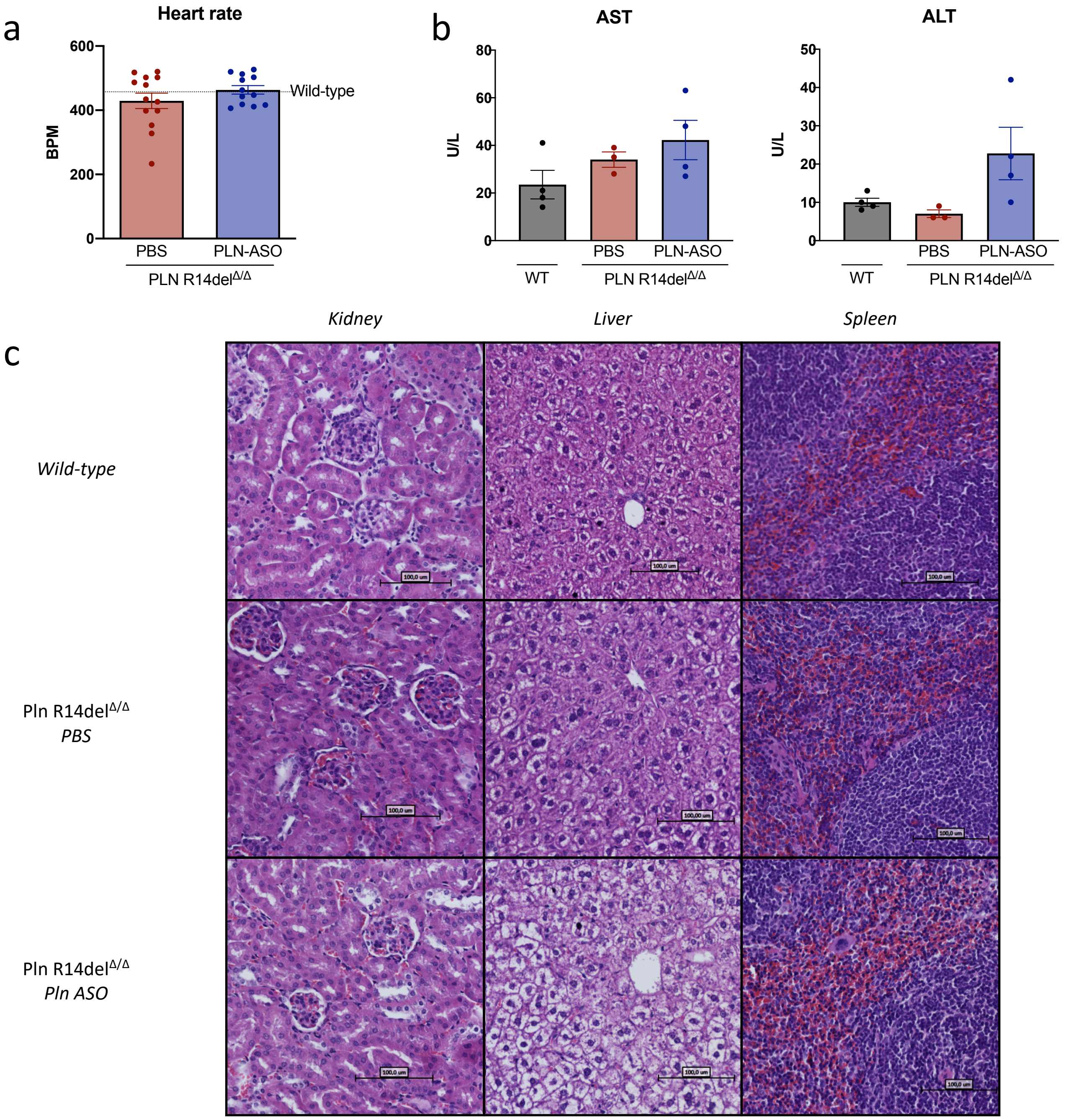

Supplementary Figure 7. No signs of increased heart rate or toxicity in PLN R14del$^{\Delta/\Delta}$ mice at T4/ 7 weeks of age

Assessment of heart rate and potential treatment toxicity in PLN R14del$^{\Delta/\Delta}$. (a) Heart rate in beats per minute (BPM) as measured by electrocardiograph, n=13 for PLN-ASO and n=14 for PBS. Wild-type data are previously published and the average is presented here for reference. (b) Plasma aspartate transaminase (AST) and alanine transaminase (ALT) measurements showing no increase in PLN-ASO treated mice compared to PBS. n=4 for wild-type and PLN-ASO and n=3 for PBS. (c) Representative H&E staining images of kidney, liver and spleen showing no signs of toxicity. This experiment has not been repeated. One-way analysis of variance and students' T-test were used for analyses, with PBS treated animals as the reference group in multiple comparison analysis. Asterix denotes significance level compared to PBS with: *P-value<0.05, **P-value<0.01, ***P-value<0.001 and ****P-value<0.0001. Single values are depicted and error bars represent standard error of the mean (SEM).

Supplementary Figure 8. RNA sequencing reveals attenuation of disease phenotype in PLN R14del$^{\Delta/\Delta}$ and long-lasting changes in the unfolded protein response results

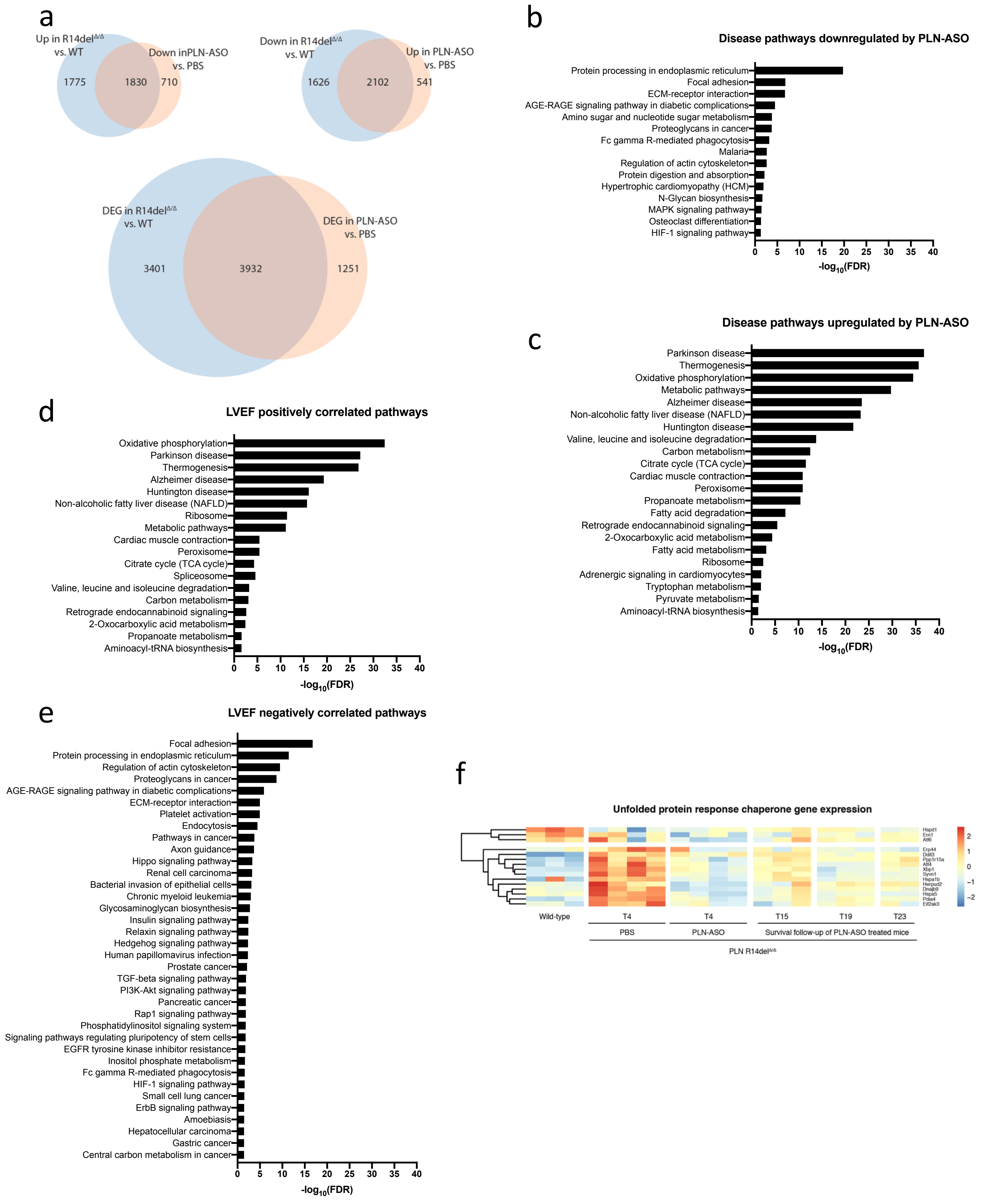

Supplementary Figure 8. RNA sequencing reveals large scale prevention of disease phenotype in PLN R14del$^{\Delta/\Delta}$ and long-lasting changes in protein processing/metabolism and the unfolded protein response results

RNA sequencing results of the PLN R14del$^{\Delta/\Delta}$ study showing (a) numbers of differentially expressed genes (DEG) in PLN R14del$^{\Delta/\Delta}$ vs. wild-type (WT, blue circles) and PLN-ASO T4 vs PBS T4 treated PLN R14del$^{\Delta/\Delta}$ mice (red circles), and their overlap ("genes reversed"). (b and c) KEGG pathways enriched in the disease specific genes down- and up-regulated by the PLN-ASO obtained from (a). (d and e) KEGG pathways enriched in genes that correlate significantly with left ventricular ejection fraction (LVEF) during the entire course of the study. (f) Heatmap showing gene expression of genes involved in the unfolded protein response (UPR), n=3 for WT, n=4 for PBS and PLN-ASO T4, n=3 for T15 and T19 and n=2 for T23.

Supplementary Figure 9. Body weight and tibia length are not affected by the PLN-ASO in PLN R14del$^{\Delta/\Delta}$ mice

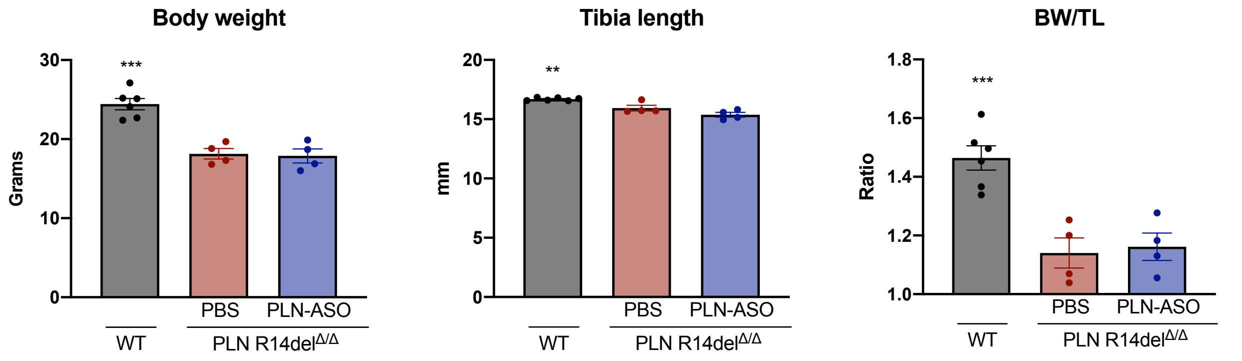

Supplementary Figure 9. Body weight and tibia length are not affected by the PLN-ASO in PLN R14del$^{\Delta/\Delta}$ mice

Body weights, tibia length and the ratio of the two (BW/TL) in PLN R14del$^{\Delta/\Delta}$ study. n=6 for wild-type, n=4 for PBS and PLN-ASO groups. One-way analysis of variance was used for analyses, with PBS treated animals as the reference group in multiple comparison analysis. Asterix denotes significance level compared to PBS with: **P*-value<0.05, ***P*-value<0.01, ****P*-value<0.001 and *****P*-value<0.0001. Single values are depicted error bars represent standard error of the mean (SEM).

Supplementary Figure 10. Detailed analyses of experimental read-outs over the course of the PLN R14del$^{\Delta/\Delta}$ study

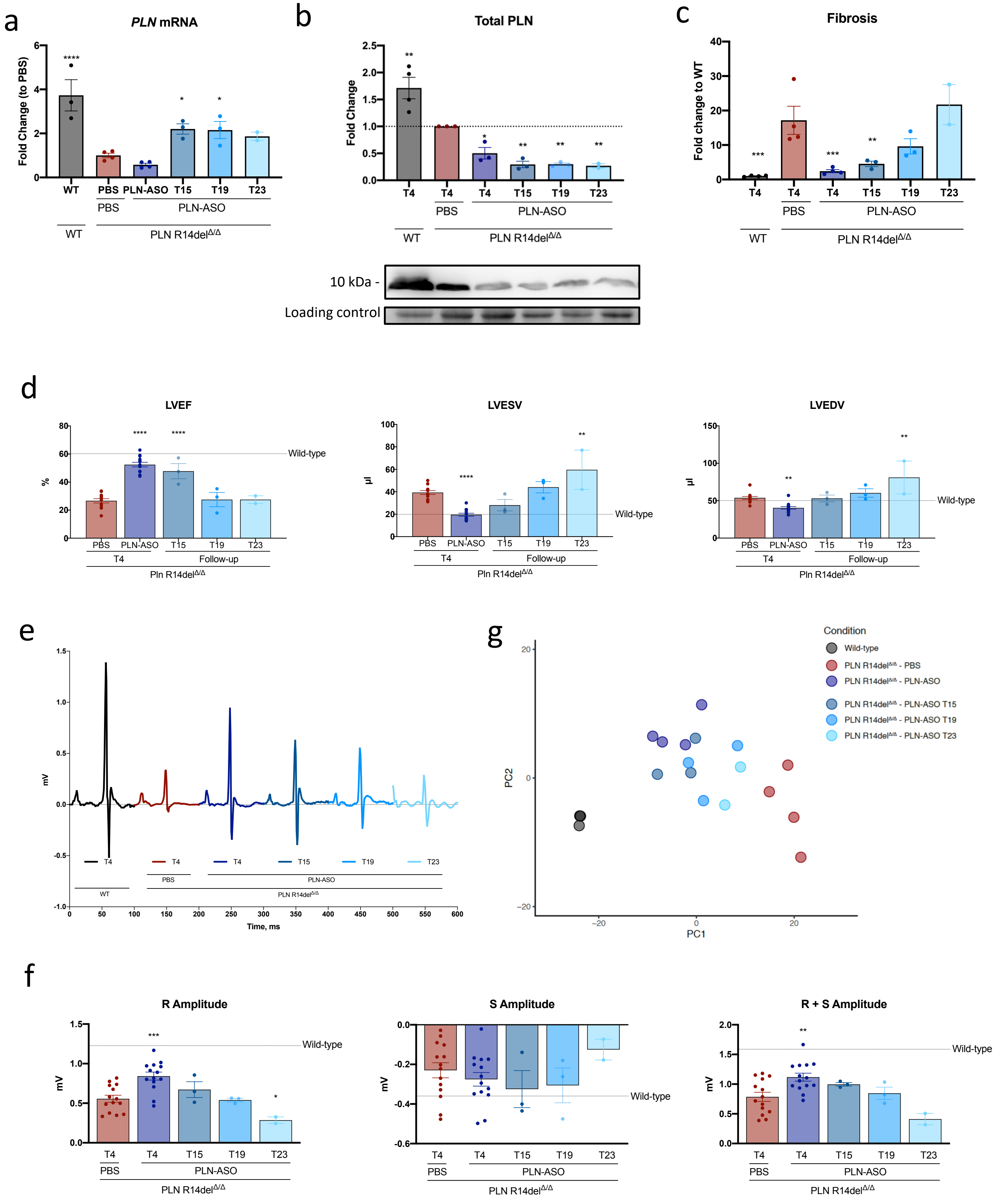

Supplementary Figure 10. Detailed analyses of experimental read-outs over the course of the PLN R14del$^{\Delta/\Delta}$ study

Full results of the entire course of the PLN R14del$^{\Delta/\Delta}$ study. Wild-type data for MRI and ECG are previously published and presented here for reference. (a) qPCR results of *Pln* mRNA expression (b) Western blot results of LV protein lysates stained for PLN protein and semi-quantified, intensities were normalized against total protein control. (c) Quantification of fibrosis based on Masson Trichrome staining whereby the blue, fibrotic area is presented as fold change in fibrotic area compared to wild-type. (d) MRI quantification of left ventricular ejection fraction (LVEF), left ventricular end systolic and end diastolic volumes (LVESV and LVEDV) (e) Representative ECG tracings of mice in different treatment groups at different time-points and quantified (f) R, S and R+S amplitude (g) Principal component analysis plot of the first 2 principal components derived from the RNA-sequencing dataset. One-way ANOVA was used for for panels a, b, c, d and f with PBS treated animals at T4 as the reference group. Asterix denotes significance level compared to PBS – T4 with: *P-value<0.05, **P-value<0.01, ***P-value<0.001 and ****P-value<0.0001 and error bars represent standard error of the mean (SEM). Number of animals for panels a, b and c: n=3 for WT and n=4 for PBS and PLN-ASO at T4, n=3 for T15 and T19 and n=2 for T23. For panel d: n=6 for WT and n=12 for PBS, n=13 for PLN-ASO at T4, n=3 for T15 and T19 and n=2 for T23. For panels e and f: n=12 for WT, n=14 for PBS, n=13 for PLN-ASO at T4, n=3 for T15 and T19 and n=2 for T23. For panel g: n=6 for WT, n=4 for PLN-ASO T4, n=3 for T15 and T19 and n=2 for T23.

Supplementary Figure 11. Full Western blot analysis of *Cspr3/Mlp*$^{-/-}$ PLN-ASO study #1 shows that PLN-ASO down-regulates PLN monomer and pentamer comparably

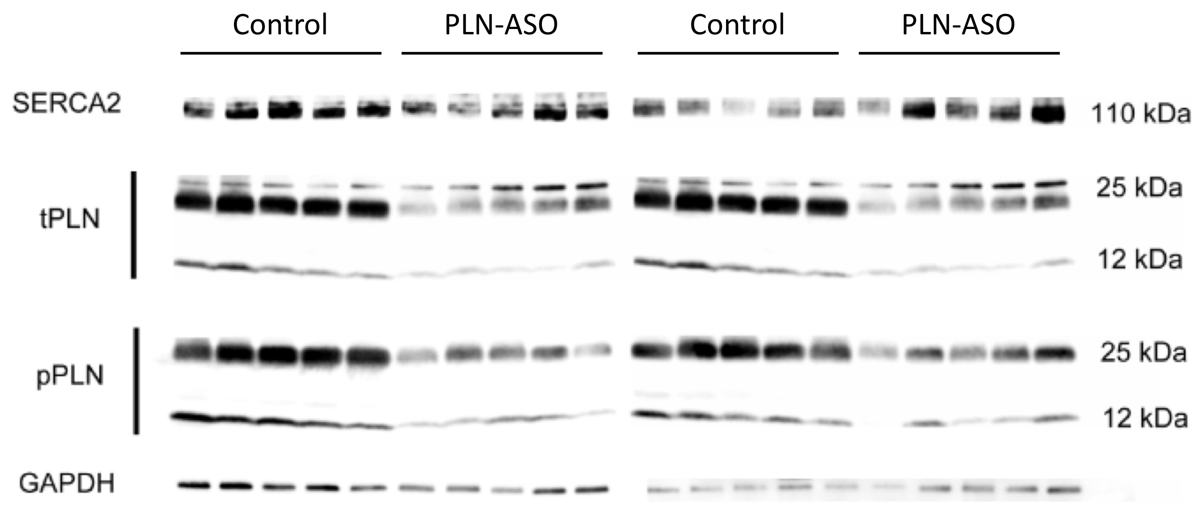

Anti-(total) PLN
2D12-MA3922

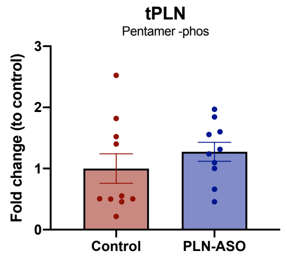

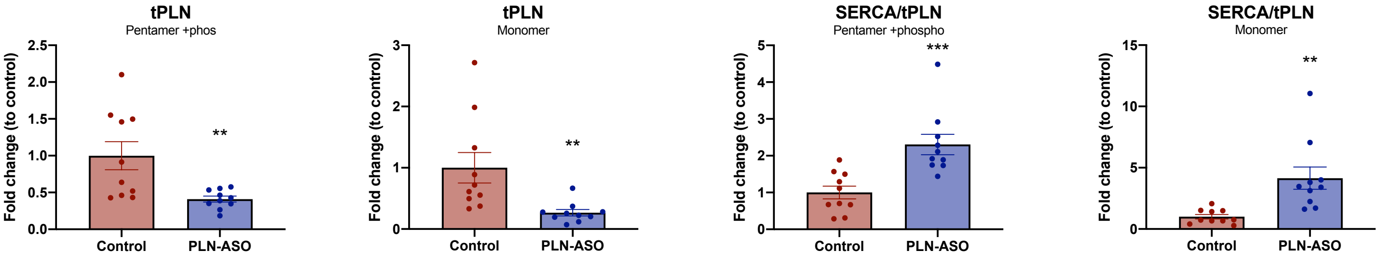

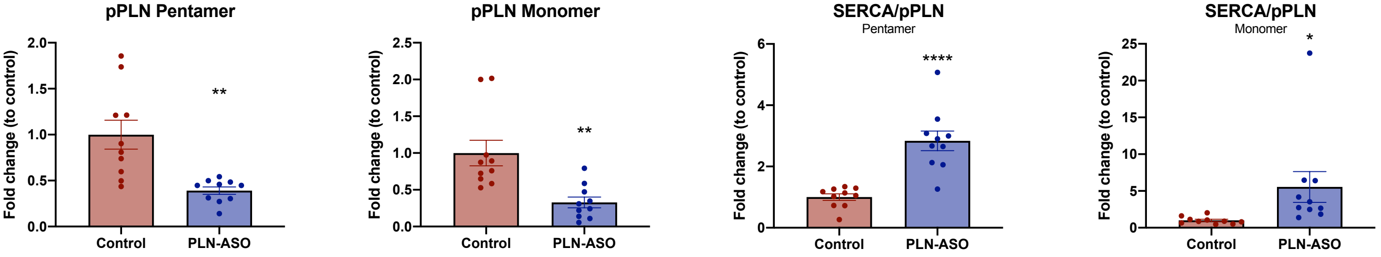

Anti-phospho-
PLN (S16+T17)

Supplementary Figure 11. Full Western blot analysis of *Cspr3/Mlp*$^{-/-}$ PLN-ASO study #1 shows that PLN-ASO down-regulates PLN monomer and pentamer comparably

Full western blot images and analysis obtained using LV protein lysates of mice in the *Cspr3/Mlp*$^{-/-}$ study. Semi-quantification of the western blots for SERCA2 and PLN monomer and pentamer, both phosphorylated or non-phosphorylated, shows similar PLN-ASO treatment effect on PLN pentamer and monomer. Control n=10, PLN-ASO n=10. Unpaired Students t-test was used for analyses. Asterix denotes significance level compared to PBS with: **P*-value<0.05, ***P*-value<0.01, ****P*-value<0.001 and *****P*-value<0.0001. Single values are depicted and error bars represent standard error of the mean (SEM). Antibodies used for PLN: rabbit anti-phospho-PLN (S16+T17), Cell signaling, mouse anti-(total) PLN 2D12-MA3922, Life Technologies.

Supplementary Figure 12. Immunohistochemistry for PLN reveals uniform downregulation of PLN protein across cardiomyocytes in *Cspr3/Mlp-/-* hearts

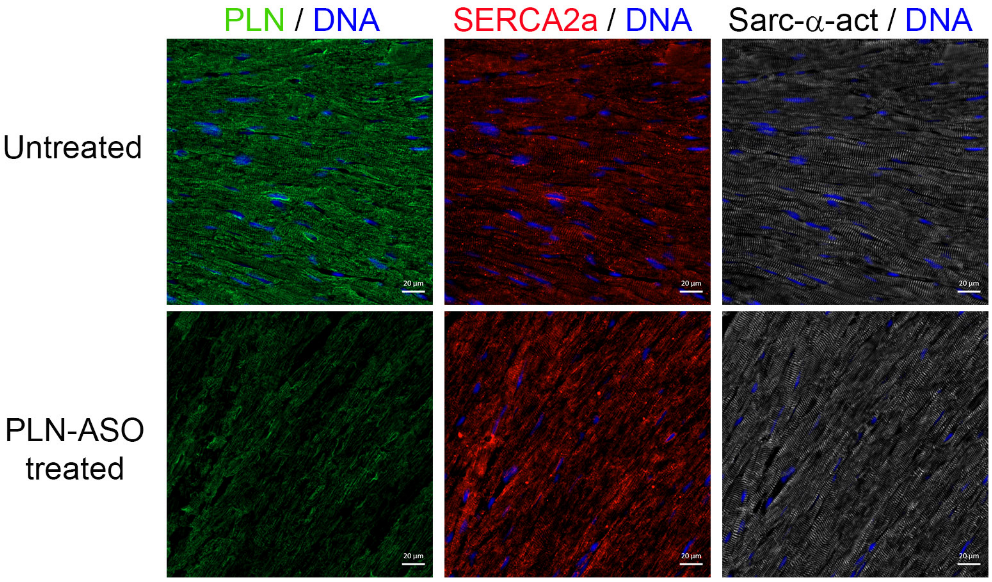

Supplementary Figure 12. Immunohistochemistry for PLN reveals uniform downregulation of PLN protein across cardiomyocytes in *Cspr3/Mlp-/-* hearts

Immunohistochemistry for PLN, SERCA2 and sarcomeric alpha-actin on cardiac sections from *Cspr3/Mlp-/-* mice, either untreated or 4 weeks after PLN-ASO treatment (dosing scheme see Figure 3a). Images include parts of the left ventricle. This experiment was not repeated.

Supplementary Figure 13. PLN protein and mRNA levels after repeated subcutaneous injections in *Cspr3/Mlp-/-* mice

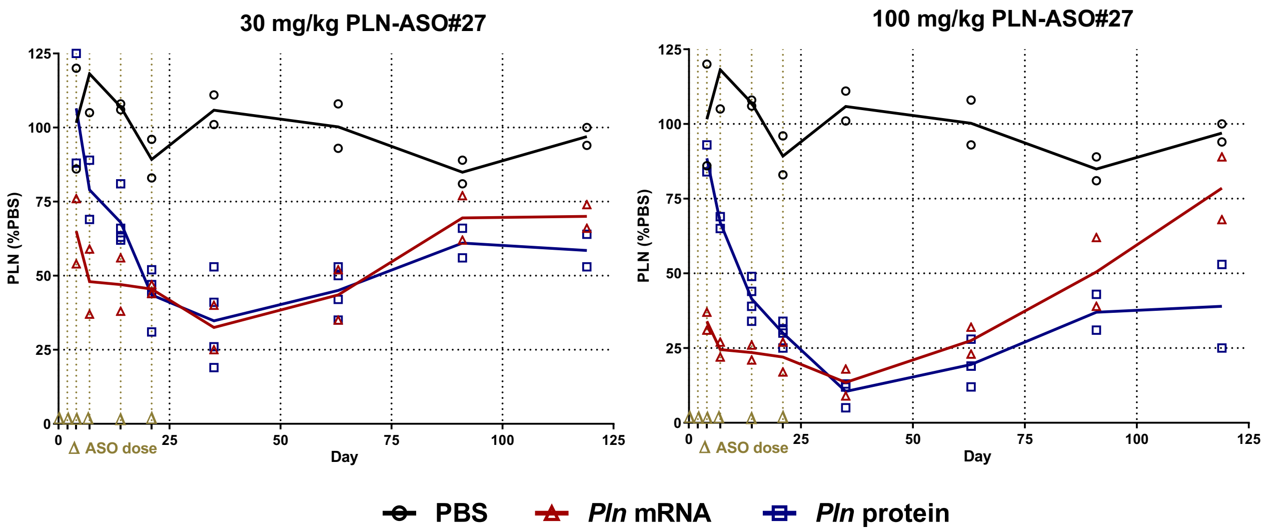

Supplementary Figure 13. PLN protein and mRNA levels after repeated subcutaneous injections in *Cspr3/Mlp-/-* mice
PLN protein and mRNA levels, depicted as % of PBS. For this study, 8 to 12-week-old female Cspr3/Mlp-/- mice were used. N = 2/time point/dose level. 30 or 100 mg/kg PLN ASO was subcutaneously injected on days 0, 2, 4, 7, 14 and 21. Cohorts euthanized on Days 4, 7, 14, 21, 35, 63, 91 and 119. Mice euthanized on Days 4, 7, 14 and 21 received a total of 2, 3, 4, and 5 ASO doses, respectively. All other cohorts (i.e. mice euthanized on Days 35, 63, 91 and 119) received 6 doses.

Supplementary Figure 14. Supplemental hemodynamic data showing increased relaxation (Tau) after PNL-ASO treatment of *Cspr3/Mlp*$^{-/-}$ mice

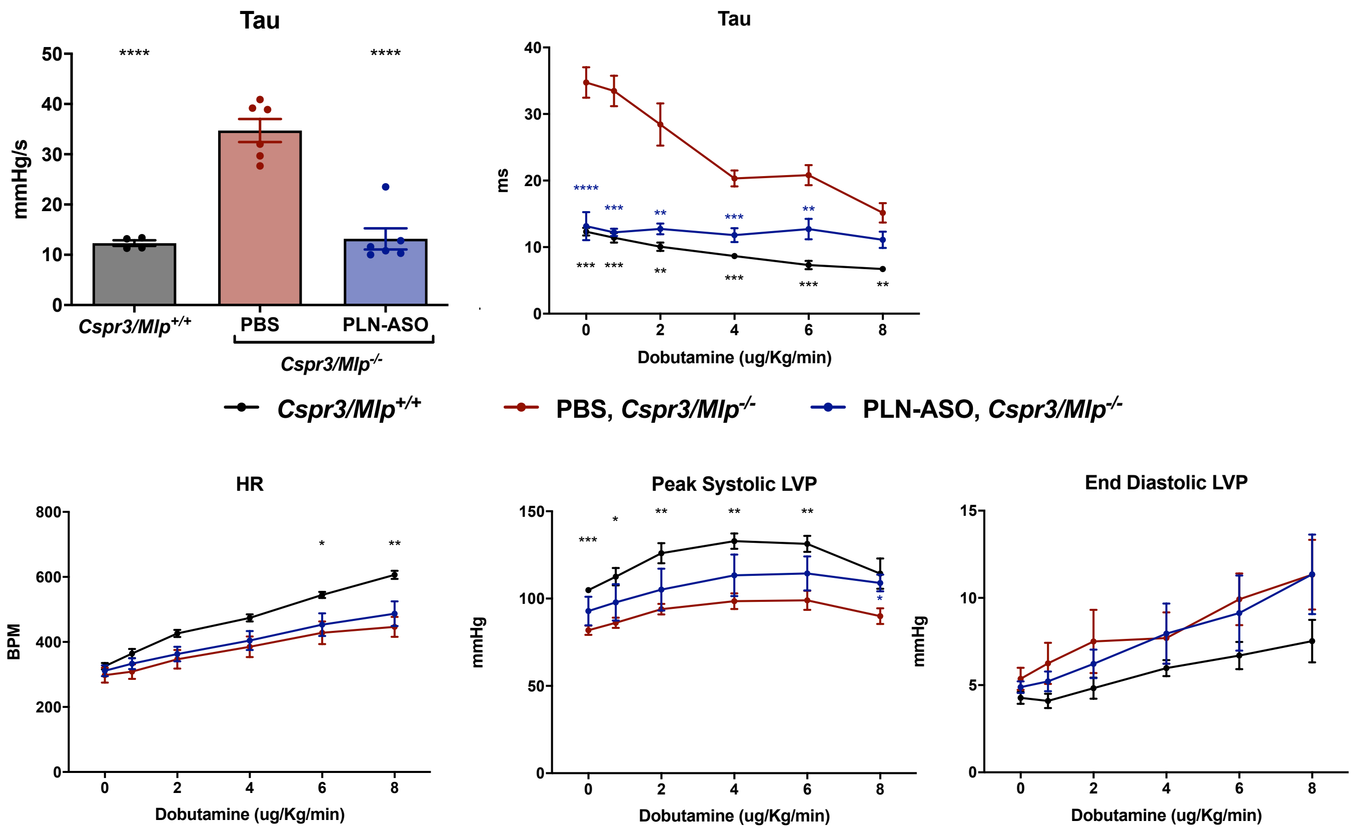

Supplementary Figure 14. Supplemental hemodynamic data showing increased relaxation (Tau) after PNL-ASO treatment of Cspr3/Mlp-/- mice
Additional hemodynamic assessment of the *Cspr3/Mlp-/-* PLN ASO study #3. Measurements of Tau, heart rate (HR) and left ventricular pressures (LVP) were performed at 24-28 days following PLN-ASO #27 or PBS treatment at baseline and upon increasing dobutamine doses of 0, 2, 4, 6, 8 µg/kg/min in a study 3 (n=4 for *Cspr3/Mlp*$^{+/+}$, n=6 *Cspr3/Mlp*$^{-/-}$ PBS and n=6 *Cspr3/Mlp*$^{-/-}$ PLN-ASO #27)$^{-}$. One and two-way analysis of variance were used for analyses under baseline and dobutamine respectively, with PBS/vehicle treated animals as the reference group in multiple comparison analysis. Asterix denotes significance level compared to PBS/vehicle control with: *<0.05, **<0.01, ***<0.001 and ****<0.0001. Single values are depicted, and error bars represent standard error of the mean (SEM).

Supplementary Figure 15. No signs of toxicity on body or heart weight in *Cspr3/Mlp*$^{-/-}$ mice treated with PLN-ASO

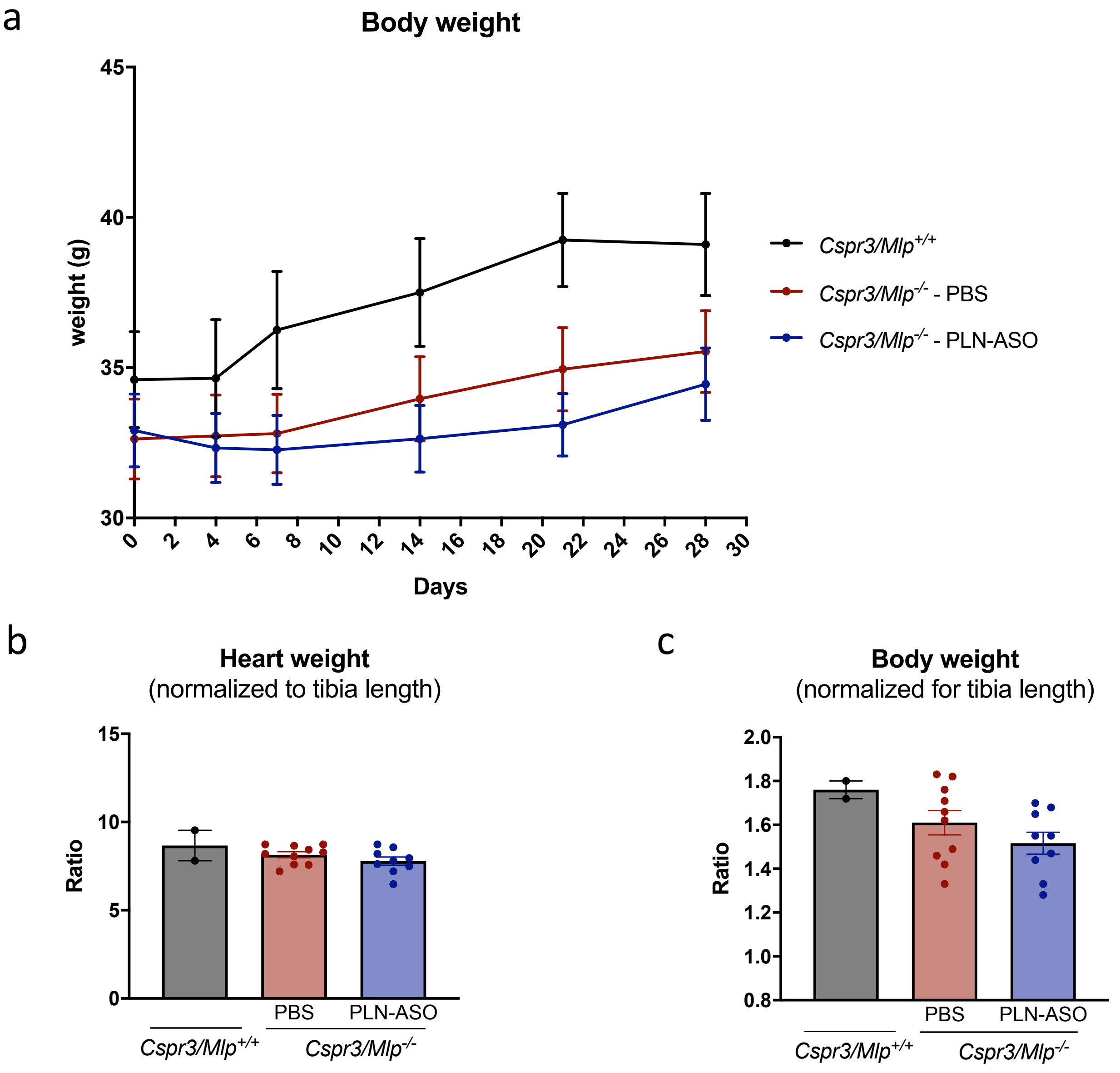

Supplementary Figure 15. No signs of toxicity on body or heart weight in *Cspr3/Mlp*$^{-/-}$ mice treated with PLN-ASO

Assessment of body weight and heart weight as toxicity read-outs in *Cspr3/Mlp-/-* study #1. No signs of toxicity were observed. a) Body weight measurements over course of study. b) Heart weight measurements normalized to tibia length. c) Body weight measurements at end of study normalized to tibia length. *Cspr3/Mlp-/-* n=10 PBS, n=10 PLN-ASO, wild-type, n=2. One and two-way analysis of variance were used for analyses, with PBS treated animals as the reference group in multiple comparison analysis, no significant differences observed. Single values are depicted, lines represent the mean, and error bars represent standard error of the mean (SEM).

Supplementary Figure 16. Increased calcium amplitude and velocity in cardiomyocytes of PLN-ASO treated *Cspr3/Mlp*$^{-/-}$ mice

* Comparison to *Cspr3/Mlp*$^{+/+}$; * Comparison to *Cspr3/Mlp*$^{-/-}$ without PLN-ASO

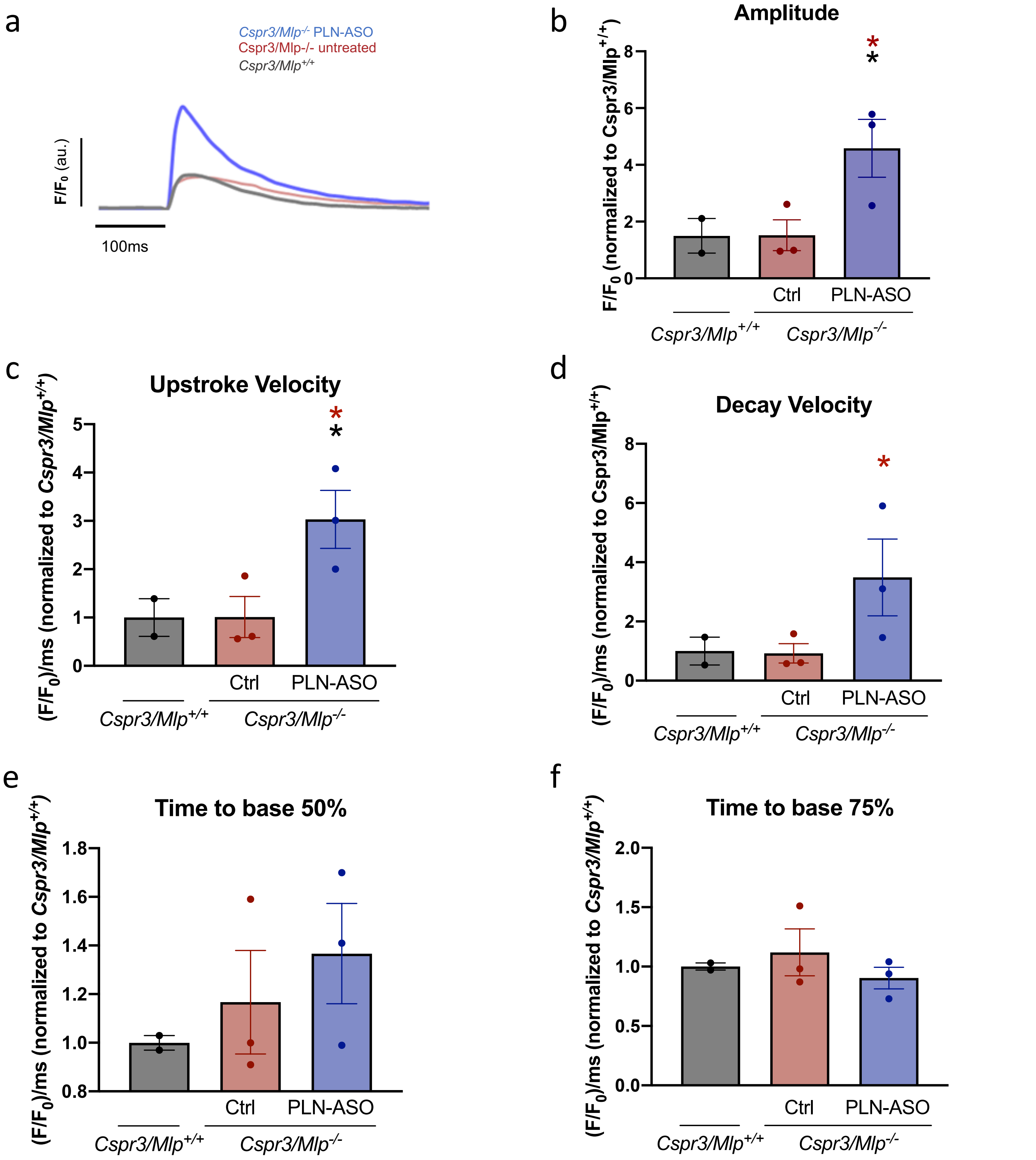

Supplementary Figure 16. Increased calcium amplitude and velocity in cardiomyocytes of PLN-ASO treated Cspr3/Mlp-/- mice

Imaging of whole cell intracellular calcium flux and analysis was performed on adult cardiomyocytes isolated 4 weeks after treatment from hearts of Cspr3/Mlp+/+, Cspr3/Mlp -/- untreated and PLN-ASO treated mice (as described in Figure 3a). Cardiomyocytes were incubated with Fluo-4 before imaging and paced at 1Hz. Calcium transients are calculated as $\Delta F/F_0$ (F_0 is background fluorescence, F is fluorescence intensity). (a) Representative traces of intracellular calcium transients comparing 3 conditions are shown. For each cell, (b) Amplitude (peak intensity), (c) Upstroke Velocity, (d) Decay Velocity, (e) Time to base 50%, and (f) Time to base 75% were calculated. PLN-ASO treatment significantly enhanced amplitude (surrogate for force), upstroke (surrogate for contraction/increased RyR2 calcium release kinetics) and decay velocity (surrogate for relaxation / SERCA2a activity) compared to untreated *Cspr3/Mlp-/-* cardiomyocytes which is in line with PLN-ASO effects observed in vivo. Total cell number measured from 3 independent experiments for *Cspr3/Mlp$^{-/-}$* and 2 for *Cspr3/Mlp$^{+/+}$*: *Cspr3/Mlp$^{+/+}$* n=286; *Cspr3/Mlp$^{-/-}$* n=146, *Cspr3/Mlp$^{-/-}$* PLN ASO n=262. Statistics: Pairwise comparisons, two-way analysis of variance was used for analyses and p-values were corrected using Tukey's procedure. Asterix denotes significance level (comparisons indicated in Figure): **P*-value<0.05, ***P*-value<0.01, ****P*-value<0.001 and *****P*-value<0.0001, error bars represent standard error of the mean (SEM).

Supplementary Figure 17. PLN-ASO vs. control ASO validation study in *Cspr3/Mlp$^{-/-}$* mice

a

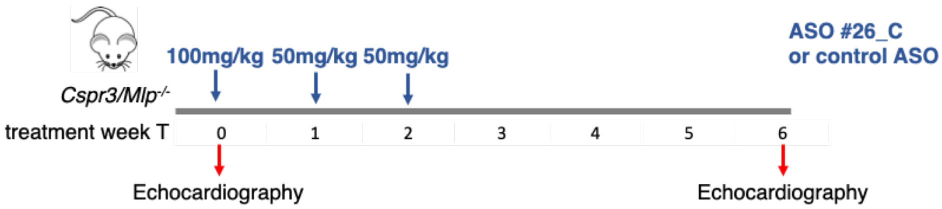

b

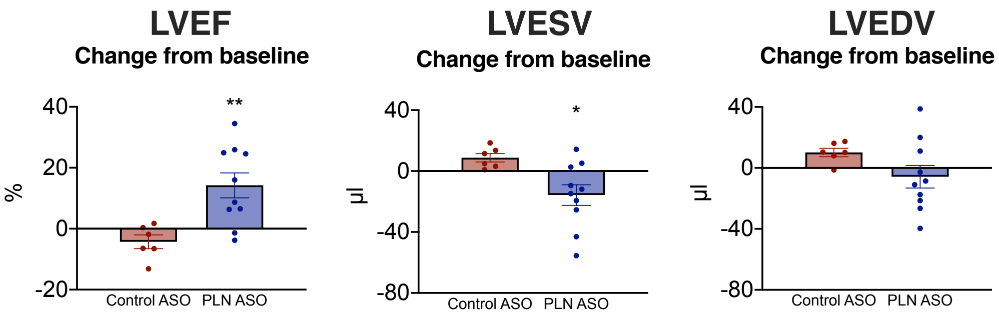

Supplementary Figure 17. PLN-ASO vs. control ASO validation study in *Cspr3/Mlp$^{-/-}$* mice
(a) Experimental design of the PLN-ASO *Cspr3/Mlp$^{-/-}$* intervention study. Study was performed with PLN-ASO #26_C (100mg/kg on day0, 50mg/kg on day7 and 14) and control ASO. Echocardiography was performed at baseline (i.e. before treatment initiation), and at end of study (i.e. after 42 days of treatment) (b) Individual echocardiography assessment and quantification of left ventricular ejection fraction [LVEF), LV end-systolic volume (LVESV), and LV end-diastolic volume (LVEDV) 28 days after treatment relative to baseline measurements. (PLN-ASO #26_C treated n=10, Control-ASO n=6). Students T test was used for analyses. Asterix denotes significance level compared to control ASO with: *<0.05, **<0.01, ***<0.001 and ****<0.0001. Single values are depicted, and error bars represent standard error of the mean (SEM).

Supplementary Figure 18. Identification of rat-specific PLN-ASO

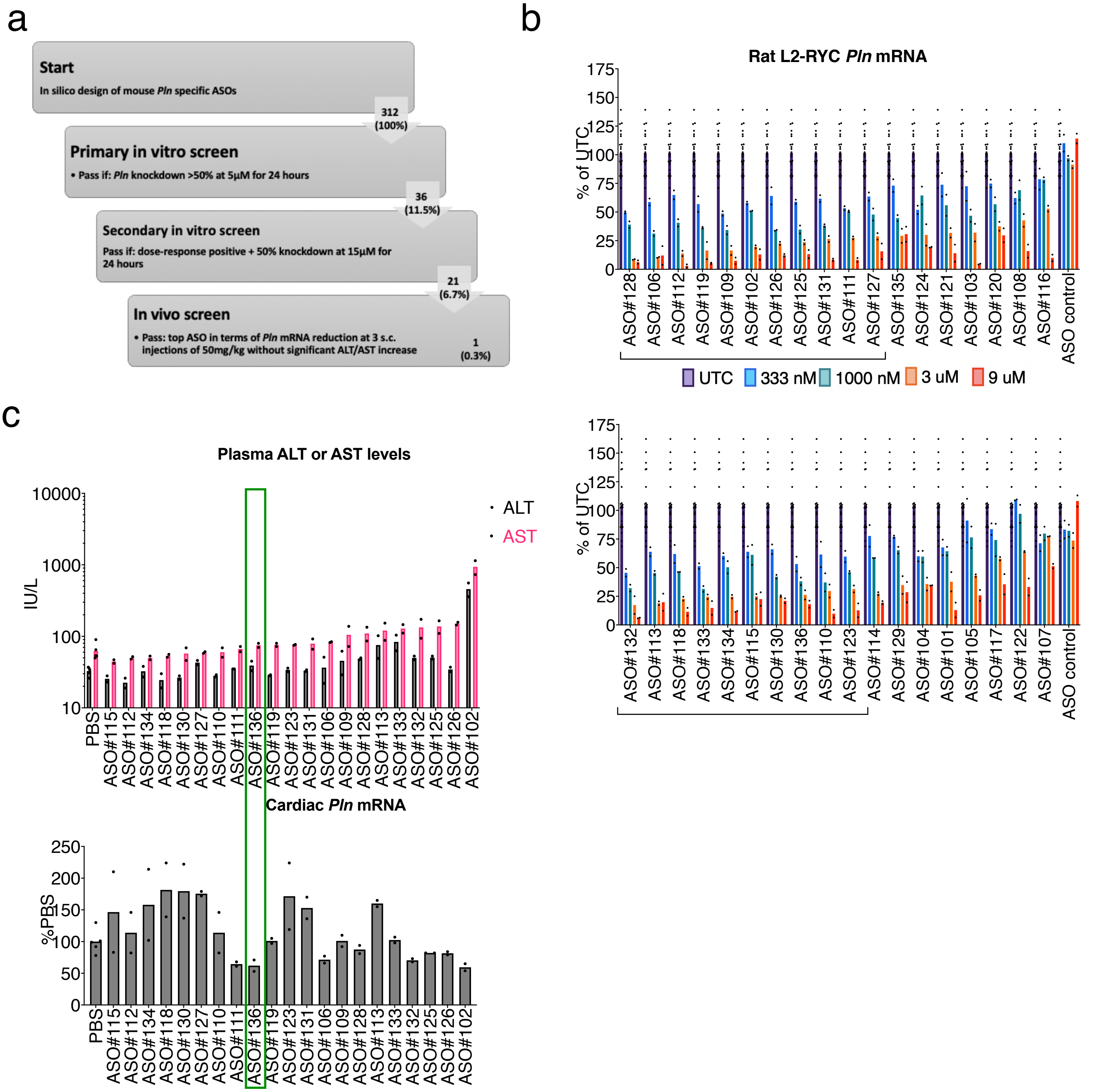

Supplementary Figure 18. Identification of rat-specific PLN-ASO

(a) Schematic illustration of experimental steps leading to identification of the rat PLN ASO lead used for rat post-MI study. (b) Results of secondary in vitro screen illustrating *Pln* mRNA reductions in rat L2-RYC cells in a dose response comparison. ASO candidates were selected (21) based on dose-responsive *Pln* mRNA reductions and ≤ ~1 mM IC50 after 24 hours of treatment following electroporation, N=24 for PBS and N=2 for each PLN ASO. (c) ASO#136 was selected as the rat PLN-ASO lead based on robust heart *Pln* mRNA reductions and plasma ALT and AST following 4 s.c. injections of 50mg/kg in healthy Sprague Dawley rats, N=4 for PBS and N=2 for each PLN ASO. Students' T-test and one-way analyses of variance were used for statistical analysis. Asterix denotes significance level compared to PBS with: **P*-value<0.05, ***P*-value<0.01, ****P*-value<0.001 and *****P*-value<0.0001.

Supplementary Figure 19. PLN-ASO dose-responsive reduction of cardiac *Pln* mRNA expression in rats after myocardial infarction

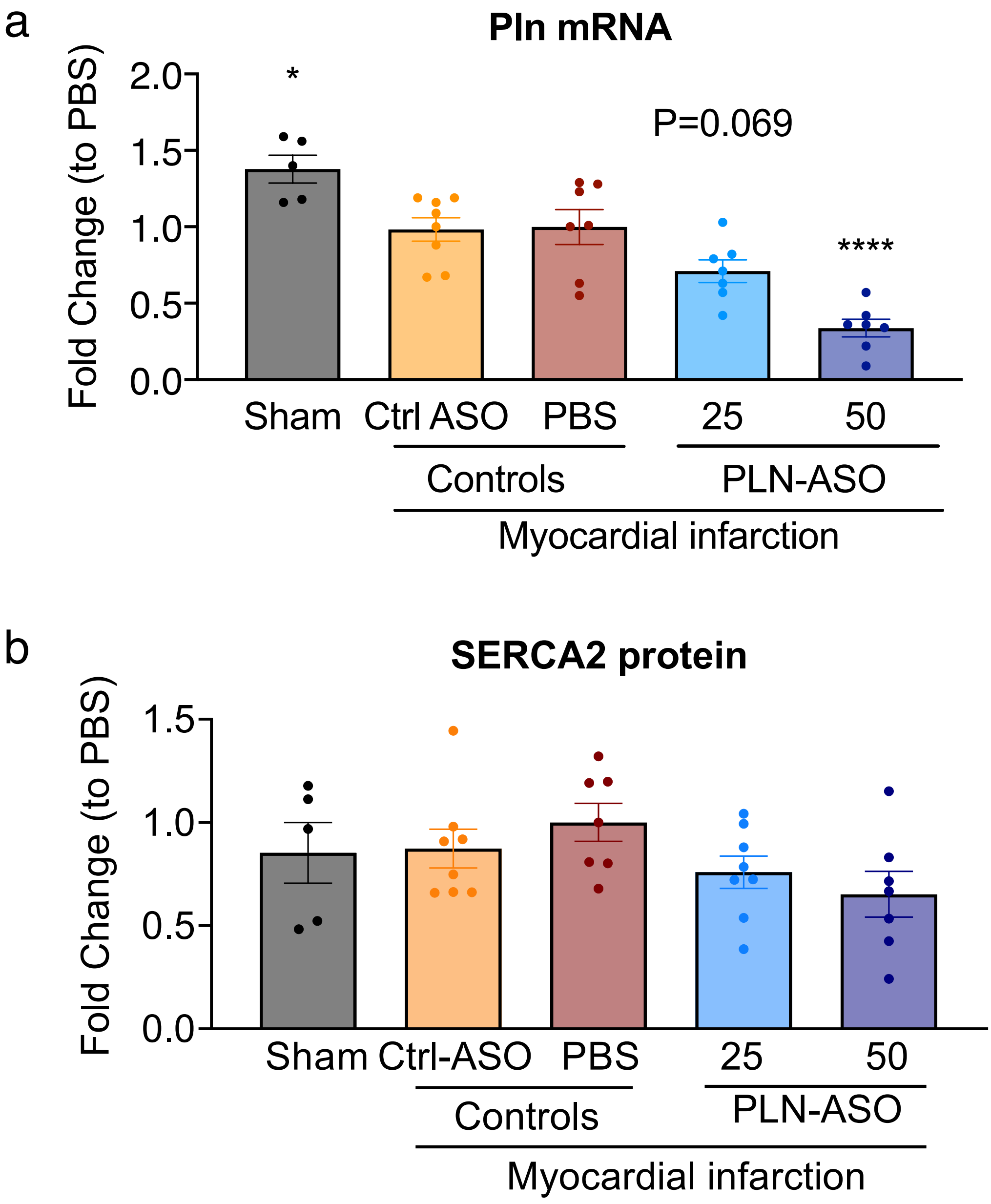

Supplementary Figure 19. PLN-ASO dose-responsive reduction of cardiac *Pln* mRNA expression in rats after myocardial infarction

(A) Results of qPCR analysis performed on heart lysates for *Pln* mRNA expression at study end (5 weeks after PLN-ASO treatment initiation). (B) Western blot results of LV protein lysates stained for PLN and SERCA2 protein and semi-quantified relative to PBS treated control samples, intensities were normalized to GAPDH. One-way analysis of variance was used for analyses, with PBS treated animals as the reference group in multiple comparison analyses. Asterix denotes significance level compared to PBS with: *<0.05, **<0.01, ***<0.001 and ****<0.0001. Single values are depicted, and error bars represent standard error of the mean (SEM). n=5 for Sham, n=7 for PBS, n=8 for Control-ASO, and n=8 PLN-ASO.

Supplementary Figure 20. Complete western blot of total PLN in rat MI study – boiled LV protein lysates

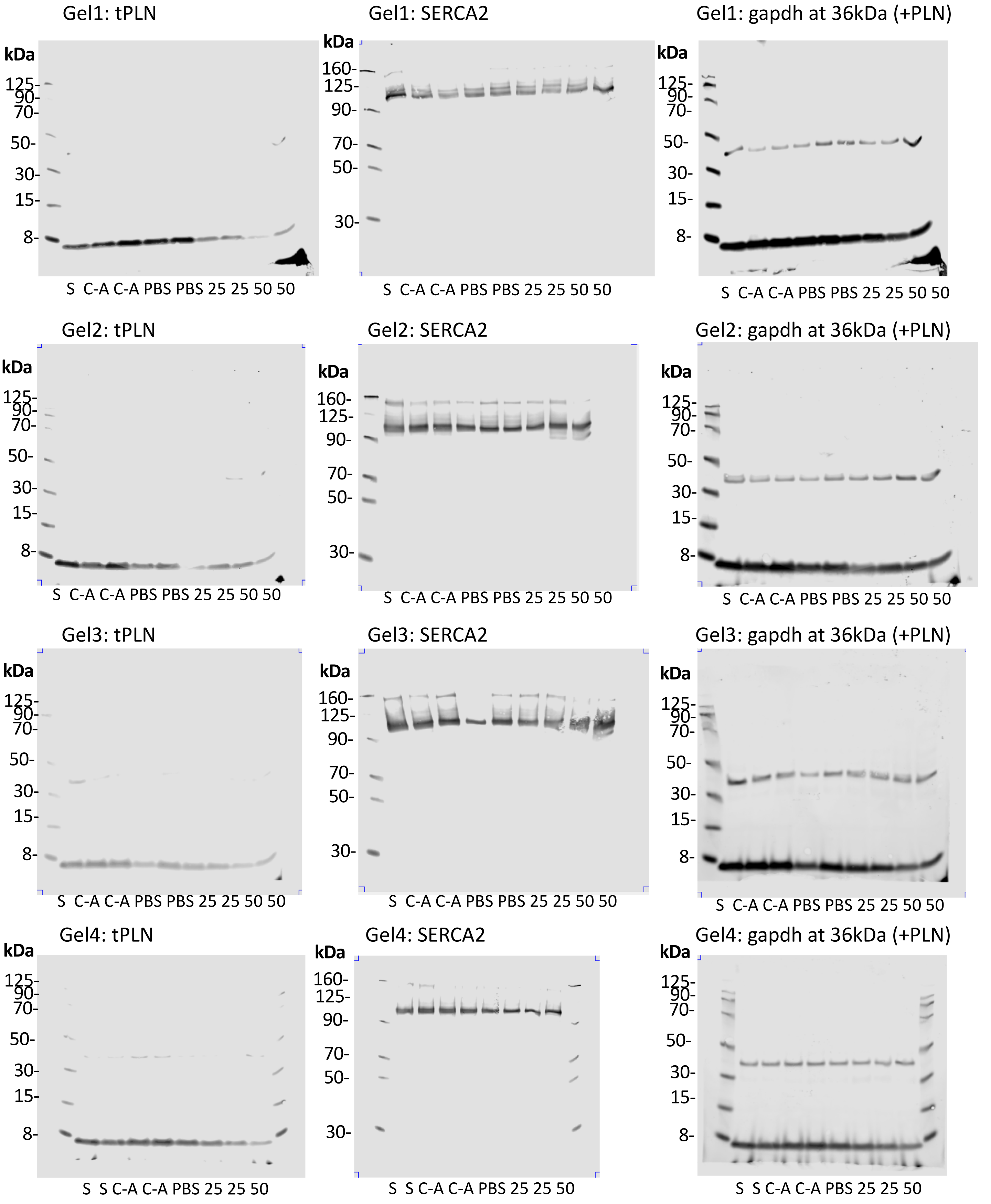

Supplementary Figure 20. Complete western blot of total PLN in rat MI study – boiled LV protein lysates

Full images of western blots performed on boiled LV protein lysates from the rat post-myocardial infarction study stained for total PLN (tPLN), SERCA2 and glyceraldehyde 3-phosphoate dehydrogenase (GAPDH) used as a protein loading control. n=5 for sham (S), n=7 for PBS, n=8 for Control-ASO (C-A), n=8 for PLN-ASO 25mg/kg, and n=7 for PLN-ASO 50mg/kg.

Supplementary Figure 21. No changes in heart rate, blood pressure or cardiac relaxation (tau) after PNL-ASO treatment of rats with a myocardial infarction

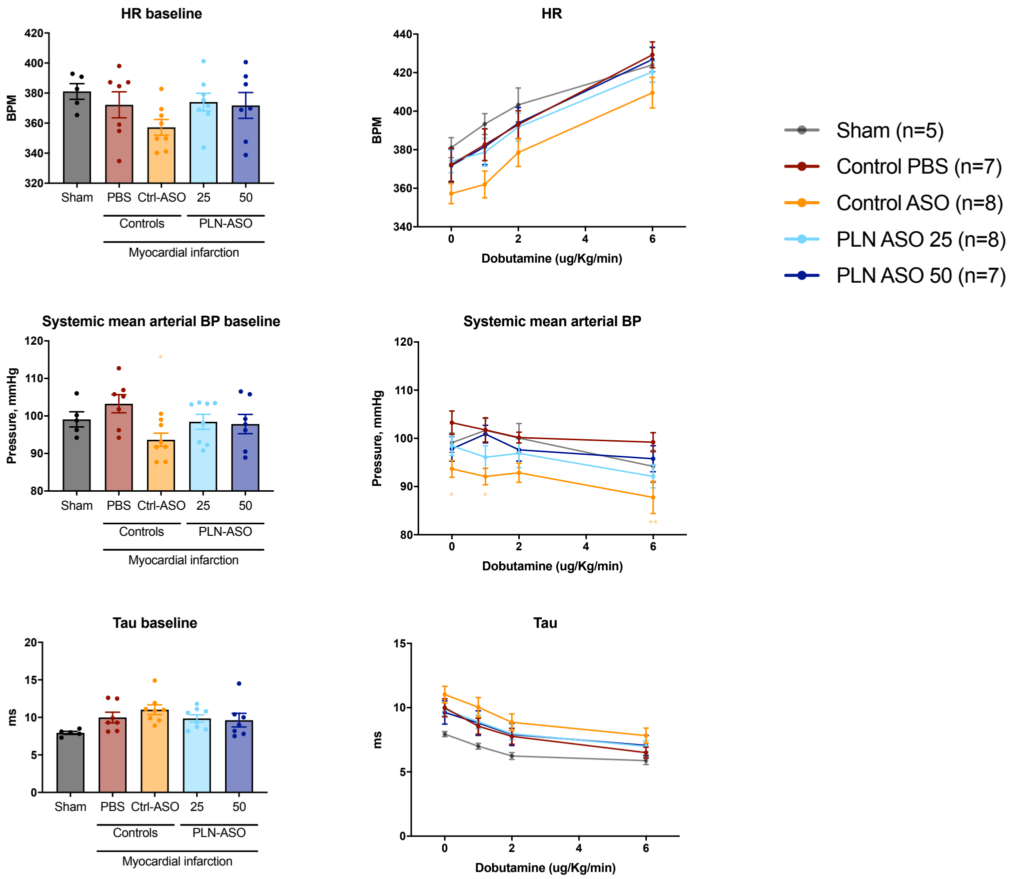

Supplementary Figure 21. No changes in heart rate, blood pressure or cardiac relaxation (tau) after PNL-ASO treatment of rats with a myocardial infarction
Full hemodynamic assessment of the rat MI PLN ASO study. Measurements of the heart rate, systemic mean arterial blood pressure (BP) and Tau were performed at study end, 5 weeks after treatment start, at baseline and upon increasing dobutamine doses of 0, 1, 2 and 6 µg/kg/min (n=5 for sham, n=7 for PBS and PLN-ASO 50mg/kg and n=8 for control ASO and PLN-ASO 25mg/kg). One and two-way analysis of variance were used for analyses at baseline and under dobutamine stress respectively, with PBS treated animals as the reference group in multiple comparison analyses. Asterix denotes significance level compared to PBS with: *P-value<0.05, **P-value<0.01, ***P-value<0.001 and ****P-value<0.0001. Single values are depicted, and error bars represent standard error of the mean (SEM).

Supplementary Figure 22. Modest reductions in body weight gain and heart weight were observed with Control-ASO in the rat MI study

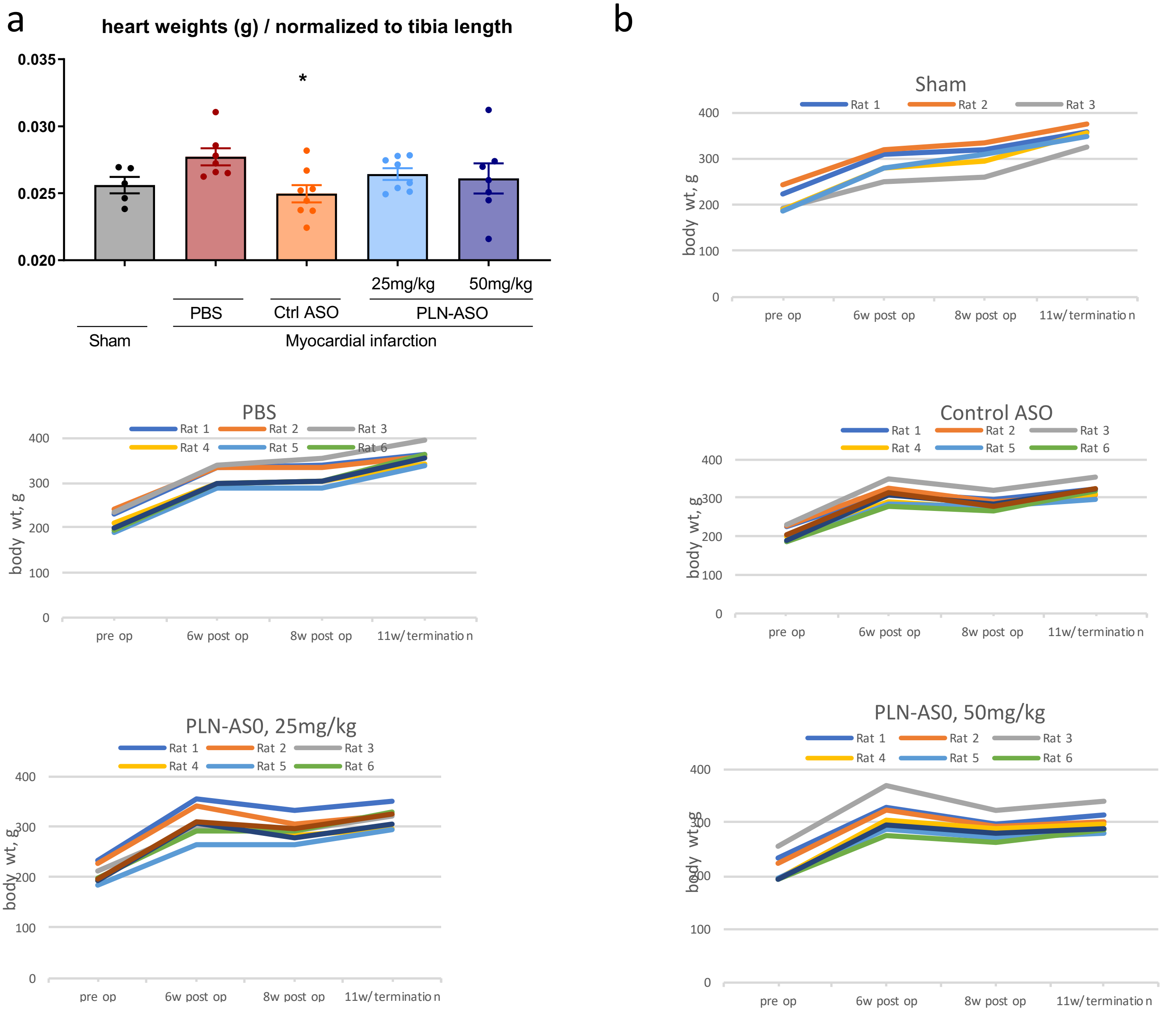

c

Comparisons between treatments and PBS at various timepoints

Treatment	Reference	Timepoint	p-value
Control ASO	PBS	6w post op	0.99
PLNASO 25mg/kg	PBS	6w post op	0.93
PLNASO 50mg/kg	PBS	6w post op	1.00
Sham	PBS	6w post op	0.040
Control ASO	PBS	8w post op	0.004
PLNASO 25mg/kg	PBS	8w post op	0.061
PLNASO 50mg/kg	PBS	8w post op	0.0009
Sham	PBS	8w post op	0.62
Control ASO	PBS	11w/termination	0.0001
PLNASO 25mg/kg	PBS	11w/termination	0.0001
PLNASO 50mg/kg	PBS	11w/termination	< 0.0001
Sham	PBS	11w/termination	1.00

Supplementary Figure 22. Modest reductions in body weight gain and heart weight were observed with Control-ASO in the rat MI study

Assessment of body weight and heart weight as toxicity read-outs in the rat MI study. a) Heart weight measurements normalized to tibia length showed no toxicity with PLN ASO treatment, but a negative with Control ASO. One-way analysis of variance was used with PBS treated animals as the reference group in multiple comparison analysis. Single values are depicted, error bars represent standard error of the mean (SEM). N=5 for sham, N=7 for PBS, N=8 for control ASO and 25mg/kg PLN ASO and N=6 for 50mg/kg PLN ASO. b) Individual body weight measurements over course of study per treatment group. Decreased body weight was observed with all ASO dosing groups (control ASO, 25 mg/kg PLN ASO, 50 mg/kg PLN ASO) relative to PBS at 11 weeks of treatments, as well as 8 weeks of treatment apart from 25 mg/kg PLN ASO. c) A linear mixed model with a random intercept was used to model body weight, including baseline, time, treatment and interaction between time and treatment; serial correlation was account for using an autoregressive-1 autocorrelation structure. Maximum likelihood estimates for the fixed effects were estimated using the nlme package (Pinheiro et al. 2019). Estimated marginal means comparing treatments at 6, 8 and 11 weeks were calculated using the emmeans package; p-values were corrected for multiple testing using Dunnett's method (Lenth 2019; Dunnett 1955). Column titles are in bold.
